# Task force of the Brazilian Society of Otology and the Brazilian Academy of Neurotology: Menière’s Disease

**DOI:** 10.1016/j.bjorl.2026.101926

**Published:** 2026-10-14

**Authors:** Henrique Furlan Pauna, Vagner Antonio Rodrigues Silva, Joel Lavinsky, Camila de Giacomo Carneiro, Lucas Resende Lucinda Mangia, Signe Schuster Grasel, Roberto Miquelino de Oliveira Beck, Márcio Cavalcante Salmito, Karen de Carvalho Lopes, Raquel Mezzalira, Paula Lobo Furtado, André L.L. Sampaio, Patrícia Mauro Mano, Cesar Bertoldo, Rogério Castro Borges de Carvalho, Lucia Joffily, Livia Nolêto de Rezende Oliveira, Pauliana Lamounier, Fabio André Selaimen, Luiz Lavinsky, Mauricio Noschang Lopes da Silva, Vanessa Mazanek Santos, Mariana Moreira de Castro Denaro, Rogério Hamerschmidt, Carlos Takahiro Chone, Arthur Menino Castilho

**Affiliations:** a Pontifícia Universidade Católica do Paraná (PUC-PR), Hospital Universitário Cajuru, Curitiba, PR, Brazil; b Universidade Estadual de Campinas (UNICAMP), Faculdade de Ciências Médicas, Department of Otolaryngology and Head and Neck Surgery, Campinas, SP, Brazil; c Universidade Federal do Rio Grande do Sul (UFRGS), Porto Alegre, RS, Brazil; d Universidade de São Paulo (USP), Faculdade de Medicina de Ribeirão Preto (FMRP), Department of Ophthalmology, Otolaryngology, and Head and Neck Surgery, Ribeirão Preto, SP, Brazil; e Universidade Federal do Paraná (UFPR), Hospital das Clínicas (HC), Curitiba, PR, Brazil; f Universidade de São Paulo (USP), Faculdade de Medicina (FM), São Paulo, SP, Brazil; g Universidade Federal de São Paulo (UNIFESP), Escola Paulista de Medicina (EPM), Division of Otolaryngology, São Paulo, SP, Brazil; h Universidade de Brasília (UnB), Faculdade de Medicina, Division of Otolaryngology, Brasília, DF, Brazil; i Clínica Borges de Carvalho Otorrinos, Rio de Janeiro, RJ, Brazil; j Universidade Federal do Estado do Rio de Janeiro (UFRJ), Hospital Universitário Gaffree e Guinle, Department of Otolaryngology, Rio de Janeiro, RJ, Brazil; k Centro Estadual de Reabilitação e Readaptação Dr Henrique Santillo (CRER), Goiânia, GO, Brazil; l Hospital de Clínicas de Porto Alegre (HCPA), Porto Alegre, RS, Brazil; m Clínica Novva, Hospital Clínica Los Angeles, Curitiba, PR, Brazil; n Hospital de Clínicas da Universidade Federal de Minas Gerais (HC/UFMG), Hearing Health and Cochlear Implant Department, Belo Horizonte, MG, Brazil

**Keywords:** Menière’s disease, Endolymphatic hydrops, Vertigo, Sensorineural hearing loss, Clinical practice guideline.

## Abstract

**Objective:**

To critically review the available evidence on the pathophysiology and diagnosis of Menière’s disease and to provide consensus-based recommendations developed by a task force of the Brazilian Society of Otology and the Brazilian Academy of Neurotology.

**Methods:**

A task force consisting of experts in otology and neurotology was established to review the scientific literature and develop thematic sections of the document. The content was discussed in both in-person and remote meetings until consensus was achieved. Recommendations were graded according to level of evidence and strength of recommendation using standardized systems for the evaluation of therapeutic interventions and diagnostic methods.

**Results:**

Menière’s disease is a heterogeneous clinical condition characterized by recurrent episodes of vertigo, fluctuating sensorineural hearing loss, tinnitus, and aural fullness. Endolymphatic hydrops represents the most consistent histopathologic finding; however, it does not alone explain the clinical expression of the disease. Current evidence indicates the involvement of multifactorial mechanisms, including genetic predisposition, immune dysfunction, inflammatory processes, and changes in inner ear ion homeostasis. Diagnosis remains primarily clinical, based on international criteria, with serial audiometry playing a key role. Electrophysiologic testing and imaging may be useful in selected situations. Clinical variability supports the concept of subphenotypes with potential diagnostic and prognostic implications.

**Conclusion:**

Menière’s disease should be recognized as a heterogeneous and multifactorial clinical entity. Diagnosis requires careful clinical evaluation and longitudinal follow-up. Advances in the understanding of pathophysiologic mechanisms and the identification of subphenotypes may contribute to more individualized diagnostic and therapeutic strategies.

## Highlights

• Menière’s disease is a heterogeneous and multifactorial inner ear disorder.
• Serial audiometry remains central to diagnosis and longitudinal follow-up.
• Endolymphatic hydrops alone does not explain disease pathophysiology.
• MRI and vestibular tests aid diagnosis, phenotyping, and prognosis.
• Personalized management should consider clinical and biologic subtypes.

## Introduction

### Historical background and evolution of the understanding of Menière’s disease

Until the mid-19th century, balance disorders were attributed exclusively to central causes, such as epilepsy or “cerebral congestion”. In 1861, Prosper Menière was the first to propose a peripheral labyrinthine origin for the symptoms, associating vertigo, hearing loss, and tinnitus with abnormalities of the inner ear.^1^ Later, in 1938, Hallpike and Cairns, in England, and Yamakawa, in Japan, described dilatation of the endolymphatic system as the characteristic histologic finding, establishing endolymphatic hydrops as the anatomic substrate of the disease.^2^^,^^3^ In the following decades, Kimura and Schuknecht, using experimental animal models, demonstrated that blockage of the endolymphatic duct or sac induced hydrops, reinforcing the role of endolymph flow in disease pathophysiology.^4^^,^^5^

Menière’s Disease (MD), considered a clinical disorder affecting the inner ear, is characterized by episodes of spontaneous vertigo, generally associated with unilateral fluctuating sensorineural hearing loss, tinnitus, and aural fullness.^1^ The disease follows an unpredictable course, with periods of remission and exacerbation, and may substantially impair patients’ quality of life.

Disease onset typically occurs between the third and fifth decades of life, with a slight female predominance. It usually begins unilaterally but may progress to bilateral involvement between 30% and 50% of cases over time. Its pathophysiology is multifactorial, involving genetic predisposition, autoimmune mechanisms, inflammatory processes, and environmental factors. The clinical course is characteristically fluctuating, and progression may result in permanent hearing deterioration and chronic vestibular dysfunction.^6^

MD can manifest in several forms. Initial vertigo may occur without auditory symptoms; alternatively, fluctuating hearing loss and tinnitus may represent the earliest manifestations. In other cases, the classic triad of unilateral hearing loss, tinnitus, and vertigo may constitute the initial presentation of the disease.^7^

### Epidemiology

Prevalence estimates vary widely, ranging from 3.5 to 513 per 100,000, although more rigorous studies and U.S. insurance claims data suggest a prevalence of approximately 190 per 100,000.^6^^,^^8^ In the United Kingdom, studies have reported an incidence of 13.1 per 100,000 person-years, with a mean age at diagnosis of 55-years.^9^ The disease occurs almost exclusively in adults, with <3% of cases arising before 18-years of age. Peak onset typically occurs between 40- and 60-years of age, and prevalence increases with advancing age. U.S. claims data show an increase in prevalence from 61 per 100,000 individuals aged 18–34 years to 440 per 100,000 individuals aged >65 years.^6^^,^^8^^,^^9^

MD is more common in women, with a female-to-male ratio ranging from 1.4:1 to 2.17:1 across studies.^6^^,^^8^, ^9^, ^10^, ^11^ It is also more prevalent among individuals of European ancestry compared with other ethnic groups.^11^^,^^12^ In the United States, approximately 615,000 individuals are estimated to be affected, with more than 45,000 new cases diagnosed annually.^12^

Patients with MD are more likely to experience migraine, depression, anxiety, sleep disorders, and immune or autonomic dysfunction compared with controls.^9^^,^^11^^,^^13^ Bilateral involvement is rare at initial presentation but may develop over time in a significant minority of patients.^6^^,^^13^ Environmental and seasonal factors, such as increased humidity and lower atmospheric pressure, may be associated with symptom exacerbation, although evidence supporting substantial seasonal variation remains limited.^10^

### Clinical diagnosis

Diagnosis should be based on symptom history and audiometric findings. Because no specific biomarker or confirmatory test exists, the diagnosis of MD requires detailed medical history, careful clinical evaluation, and exclusion of other vestibular and non-vestibular etiologies. In 2015, the Bárány Society revised the diagnostic criteria and established the following classifications^6^:

1) Definite MD: Two or more spontaneous episodes of vertigo, each lasting 20 min to 12 h; Audiometrically documented low- to mid-frequency sensorineural hearing loss in one ear on at least one occasion; Fluctuating aural symptoms in the affected ear; and Not better accounted for by another vestibular diagnosis.
2) Probable MD: Two or more episodes of vertigo or dizziness, each lasting 20 min to 24 h; Fluctuating aural symptoms in the affected ear; and Not better accounted for by another vestibular diagnosis.

Hearing loss is a required criterion for the diagnosis of MD. Low-frequency sensorineural hearing loss is defined as increases in pure-tone bone-conduction thresholds of at least 30 dB HL at each of 2 contiguous frequencies below 2000 Hz in the affected ear.^6^

Given the variability in clinical presentation, it is important to note that establishing a complete and accurate diagnosis may take several months. There is consensus that a failure of inner ear homeostasis underlies the disease. Endolymphatic hydrops alone does not fully explain the clinical features, hearing loss progression, or frequency of vertigo attacks. More recent studies have suggested that hydrops is not the cause, but rather a response to endolymphatic sac dysfunction. The mechanisms leading to hearing loss and episodic vertigo remain elusive.^14^

### Endolymphatic hydrops and histologic changes

Endolymphatic hydrops is considered the hallmark histopathologic finding of MD and has been consistently observed in classic temporal bone studies.^15^^,^^16^ Although hydrops has been frequently associated with the disease, debate persists as to whether it represents a primary cause or a consequence of the pathophysiologic process.^17^ These investigations underscore the importance of histologic analysis in understanding the disease, including the assessment of the endolymphatic sac and aqueduct, as well as of cochlear and vestibular cells.

Endolymphatic hydrops is present in nearly all temporal bones from patients with MD but may also occur secondarily in otosclerosis, trauma, chronic otitis media, or autoimmune diseases.^16^^,^^18^ Histologically, atrophy of the stria vascularis, degeneration of the spiral ligament, and loss of fibroblasts are observed, compromising potassium recycling and the endocochlear potential.^16^^,^^18^

Outer sulcus cells exhibit morphologic changes relevant to ion homeostasis,^19^ whereas the vestibule shows hair cell degeneration, synaptic abnormalities, and changes in supporting cells.^20^ Modern animal models have shown that combining endolymphatic sac electrocauterization with desmopressin administration induces severe hydrops and vertigo attacks, reinforcing the role of vasopressin in regulating inner ear fluid homeostasis.^21^

### Endolymphatic duct, sac, and drainage system

The endolymphatic sac plays a key role in the ionic and volume regulation of the endolymph. Histologic studies have demonstrated epithelial degeneration and hypoplasia associated with loss of mineralocorticoid-regulated transport proteins (ENaC, NKA, NCC, and SGK1), suggesting impaired ion homeostasis.^22^^,^^23^ Recent evidence further demonstrates that β1- and β2-adrenergic stimulation of the sac epithelium promotes active electrogenic transport, suggesting that the autonomic nervous system may modulate the regulation of the endolymph and provide potential therapeutic targets.^24^

The ductus reuniens, the anatomic connection between the cochlea and vestibule, has recently been visualized in 3D using high-resolution micro-Computed Tomography (CT) and appears as a narrow structure with areas of luminal stenosis that may function as blockages impeding endolymph flow.^25^ Changes in the patency of this duct may explain abnormal communication between cochlear and vestibular compartments in patients with hydrops.

Three-dimensional reconstructions of the drainage system have also shown volumetric remodeling of the membranous labyrinth in patients with MD, including dysfunction of Bast’s valve and blockage of the longitudinal flow of endolymph, findings confirmed in temporal bone studies.^26^^,^^27^

Regarding endolymphatic sac surgery, histologic analyses have shown the persistence of hydrops in all cases even after decompression, despite some patient reports of clinical improvement in vertigo symptoms, suggesting that the benefit does not depend on the morphologic resolution of a distended membranous labyrinth.^28^

### Blood-labyrinth barrier and ion homeostasis

Disruption of the blood-labyrinth barrier contributes to osmotic instability and hydrops formation. Impaired activity of the epithelial sodium and potassium channels, associated with loss of aldosterone-sensitive pathways, can compromise the electrolyte balance of the endolymph.^22^^,^^23^ Furthermore, vasopressin acting through V2 receptors and aquaporin channels increases water retention, thereby exacerbating the distension of the membranous labyrinth.^21^

### Secondary hydrops and anatomic variations

Endolymphatic hydrops may develop secondary to multiple disorders, including otosclerosis, trauma, chronic otitis media, and autoimmune diseases.^27^^,^^29^ Histologic and radiologic studies have also shown that vestibular aqueduct morphology correlates with endolymphatic sac abnormalities, predisposing certain individuals to unilateral forms of the disease.^30^

### Autoimmunity

A significant subset of patients shows evidence of autoimmunity. Studies have demonstrated abnormal reactivity against inner ear proteins, association with systemic autoimmune diseases, and favorable response to corticosteroids and immunosuppressive therapy.^30^ Reports of response to biologic agents such as etanercept further suggest an autoimmune etiology in a subset of cases.^31^

### Genetics

MD has a complex, multifactorial molecular pathogenesis involving immune dysregulation, genetic susceptibility, epigenetic changes, and abnormal ion homeostasis. Approximately 5%–15% of patients exhibit an inheritance pattern.^32^^,^^33^ Several genes associated with extracellular matrix composition and stereocilia adhesion have been implicated, including OTOG, MYO7A, TECTA, CDH23, PCDH15, and ADGRV1.^34^ Variants in the COCH gene have also been described in disease-like phenotypes.^35^

Recent studies have replicated rare OTOG variants in Brazilian patients, confirming previous findings in Spanish cohorts and suggesting that this gene may play a major role in MD pathophysiology.^36^ Genes related to ion channels and epithelial integrity, such as ATP1A1, ATP1B2, KCNJ10, KCNQ1, and CLDN11, have also emerged as candidate genes.^37^, ^38^, ^39^

Patient stratification may involve shared clinical characteristics (phenotypes) or histologic/radiologic characteristics (endotypes). Increasing evidence suggests that MD is not a single disease entity, but rather a clinical condition encompassing multiple underlying pathophysiologic mechanisms, including genetic predisposition in a substantial proportion of cases. Studies of affected families have identified inherited forms of the disease, showing an autosomal dominant pattern of inheritance with variable penetrance. Mutations have been described in genes such as FAM136A, DTNA, PRKCB, SEMA3D, OTOG, and TECTA, all of which are associated with structural and molecular functions of the inner ear, especially ion homeostasis, cell adhesion, and tectorial membrane structure.^40^^,^^41^

### Molecular biology

Recent evidence implicates autoinflammatory mechanisms, particularly activation of the NLRP3 inflammasome in macrophage-like vestibular cells, which is associated with downregulation of Serum/Glucocorticoid-regulated Kinase-1 (SGK1). SGK1 normally inhibits NLRP3 assembly and activation; its deficiency results in increased Interleukin (IL)-1β production, inner hair cell damage, and exacerbation of audiovestibular symptoms. Pharmacologic inhibition of SGK1 has increased disease severity, whereas NLRP3 inflammasome blockade has improved symptoms in murine models, highlighting the key role of innate immune activation in MD pathogenesis.^42^

Genomic and epigenomic studies have identified substantial heterogeneity among patients. Genome-wide DNA methylation profiling has revealed 3 distinct molecular subgroups of MD, each characterized by unique patterns of gene enrichment related to cytokine signaling, T-cell and Natural Killer (NK)-cell pathways, and neuronal function. The KDMB4 gene has emerged as a key locus with altered DNA accessibility and methylation across multiple subgroups, suggesting a role in disease susceptibility and immune regulation.^43^

Bioinformatics and machine-learning approaches have identified hundreds of genes associated with MD, with enrichment in immune response pathways. Notably, CD5 and AJUBA have been proposed as potential biomarkers, while immune cell profiling implicates resting CD4 T-cells, gamma-delta T-cells, and activated dendritic cells as major cellular mediators. Mendelian randomization analyses further support a causal relationship between immune cell dysregulation and disease development.^44^

Genetic studies have identified rare variants in genes encoding structural proteins of the inner ear (TECTA, MYO7A, OTOG, CDH23, PCDH15, and ADGRV1), suggesting that defects in stereocilia integrity and their interaction with the tectorial and otolithic membranes may contribute to familial forms of the disease. Additionally, polymorphisms in ion channel and transporter genes (SIK1, SLC8A1, KCNE1, and KCNE3) have been associated with altered potassium, sodium, and calcium homeostasis, potentially predisposing to endolymphatic hydrops, a hallmark of MD.^45^, ^46^, ^47^

Immunologic profiling of peripheral blood mononuclear cells in affected patients shows upregulation of genes such as GSTM1 and TMEM176A/B, along with downregulation of SLC4A1/10, further supporting the role of immune dysfunction and altered ion transport in disease pathogenesis.^48^ Elevated levels of proinflammatory cytokines (IL-1β, IL-6, Tumor Necrosis Factor alpha [TNFα], and macrophage Migration Inhibitory Factor [MIF]) and increased NK cell activity have also been observed, findings consistent with an autoinflammatory rather than a classic autoimmune process.^47^^,^^49^

MD has been associated with multiple pathophysiologic mechanisms involving interactions among genetic, immunologic, and structural factors. Current evidence suggests that both autoimmune and autoinflammatory mechanisms may contribute to different disease subtypes. Evidence indicates that innate immune activation, mediated by the NLRP3 inflammasome and cytokine cascade release, plays a key role in local inflammation and tissue damage.^42^^,^^47^^,^^49^

Furthermore, genetic and epigenetic heterogeneity affecting pathways related to immune response, neuronal function, and ion transport contributes to the clinical variability observed among patients.^43^^,^^45^, ^46^, ^47^ Structural variants in genes encoding proteins responsible for stereocilia and cell membrane integrity have also been described, compromising cochlear mechano-electrical transduction.^45^ Dysregulation of ion channels and transporters in the endolymph may culminate in endolymphatic hydrops,^46^^,^^47^ whereas abnormalities in peripheral immune cell profiles and cytokine expression suggest a systemic immune imbalance associated with MD.^44^^,^^47^^,^^48^

### Clinical subphenotypes and diagnostic implications

Clinical heterogeneity has led to the proposal of classifications based on phenotypes and endotypes. Subtyping may consider predominant manifestations (cochlear vs. vestibular), presumed etiology (genetic, autoimmune, or viral), or histopathologic patterns of the endolymphatic sac.^32^^,^^34^

MD has marked clinical heterogeneity, motivating the description of distinct phenotypes based on predominant symptom characteristics, patterns of involvement, and genetic features. Recognition of these phenotypes is clinically important, as it may influence prognosis, treatment strategies, and the understanding of the pathophysiologic basis of the disease.

Genetics may provide a pathway for identifying shared mechanisms within a clinically heterogeneous population. However, identifying the genetic causes of MD remains challenging because of its complex and multifactorial nature. Genetic analysis represents a promising, although still evolving, tool for patient stratification.^33^

Frejo et al.^33^ proposed 5 clinical subtypes of MD based on phenotypic characteristics, family history, and associated comorbidities: (1) Classic MD; (2) Delayed MD – onset after 60-years of age without family history; (3) Familial MD – positive family history; (4) Migraine-associated MD; (5) Autoimmune MD – presence of autoimmune comorbidities (e.g., systemic lupus erythematosus, Hashimoto’s thyroiditis).

According to the authors, in a cohort of 398 patients evaluated using 2-step cluster analysis, classic MD was the most prevalent subtype (46% of patients), characterized by progressive sensorineural hearing loss without a history of migraine or autoimmune comorbidities. Delayed MD accounted for 17% of patients, whereas familial MD represented approximately 13%. Types 4 and 5, each comprising slightly more than 10% of patients, included those with a history of migraine and concomitant autoimmune disorders, respectively.

Future perspectives extend beyond clinical observation. It is essential to validate and integrate diagnostic tools to enable a precision medicine approach to patient care, ultimately transforming the understanding and management of MD.

From a diagnostic perspective, new methods are under investigation. Electrovestibulography (EVestG) has demonstrated neuronal firing patterns specific to MD, with sensitivity of 75% and specificity of 80% in preliminary studies.^37^ High-resolution Magnetic Resonance Imaging (MRI), with a 3D Constructive Interference in Steady State (3D-CISS) sequence, has revealed signal abnormalities in labyrinthine structures correlated with the symptomatic side, although definitive diagnostic value has not yet been established.^38^ In addition, techniques for vestibular function mapping have been tested in patients with unilateral peripheral deficits, allowing the assessment of dysfunction topography.^50^

Recommendations for the diagnosis and pathophysiology of Menière’s disease

I The clinical diagnosis of MD should be based on the revised Bárány Society criteria (2015), combining recurrent vertigo, fluctuating sensorineural hearing loss, and associated auditory symptoms after exclusion of other causes. (Strength of recommendation: strong; Level of evidence: high).
II Endolymphatic hydrops should be recognized as the hallmark histopathologic finding of MD, although it is insufficient to independently explain the pathophysiology of the disease. (Strength of recommendation: moderate; Level of evidence: moderate).
III MD is a heterogeneous and multifactorial condition that may involve genetic, immunologic, inflammatory, and ion regulatory mechanisms. (Strength of recommendation: strong; Level of evidence: moderate).
IV Genetic predisposition or autoimmunity should be investigated in patients with bilateral disease, early onset, positive family history, or favorable response to corticosteroids and immunosuppressive therapy. (Strength of recommendation: moderate; Level of evidence: low).

### Differential diagnoses

MD is a clinical syndrome characterized by recurrent episodes of spontaneous vertigo. Owing to symptom overlap, it is essential to include MD in the differential diagnosis of other episodic vestibular disorders.

Vestibular migraine represents the main differential diagnosis of MD. Both conditions may present with spontaneous vertigo attacks. Although auditory symptoms are traditionally associated with MD, studies indicate that they may also occur in vestibular migraine. However, the low-frequency hearing loss typical of MD is generally not expected in vestibular migraine.^7^ A history of characteristic migraine headaches and symptoms, such as photophobia and phonophobia, may also assist in differentiation. Nevertheless, these manifestations do not always coincide with vestibular episodes, potentially leading to misdiagnosis. Furthermore, migraine may coexist with MD, either as a comorbidity or as a specific clinical phenotype known as migraine-associated MD.^33^^,^^51^

Other episodic vestibular disorders, including Benign Paroxysmal Positional Vertigo (BPPV), third-window syndrome, vestibular paroxysmia, and motion sickness, should also be considered in the differential diagnosis. Characteristics such as attack duration, associated symptoms, and triggering factors are useful in distinguishing among these conditions.^7^^,^^52^ Moreover, episodes of dizziness of non-vestibular origin, such as orthostatic intolerance, panic disorders, and heart diseases, can be differentiated on the basis of these features.^52^

Diseases associated with vestibular symptoms and hearing loss, although less prevalent, should not be overlooked.^7^ These include central vestibular disorders, such as tumors, multiple sclerosis, and vascular disorders, as well as immune-mediated diseases, ototoxicity, neurosyphilis, Lyme disease, and Cogan syndrome.

The diagnosis of MD requires observation of recurrent attacks with characteristic features. In early presentations, especially in urgent and emergency settings, the disease should be considered among other possible diagnoses, including posterior fossa stroke, transient ischemic attacks, vestibular neuritis, acute labyrinthitis, vestibular migraine, and BPPV. A detailed medical history and comprehensive physical examination, with emphasis on the HINTS battery (head impulse test, nystagmus, and test of skew), are essential to clinical reasoning in this context.^52^^,^^53^ Importantly, the presence of hearing loss concomitant with an acute vestibular syndrome should initially prompt investigation for a possible central vestibular disorder, even if subsequent follow-up ultimately reveals an initial MD attack.^54^

Although still controversial, several comorbidities may influence the course of MD and, when present, should be appropriately managed to optimize disease control. These include autoimmune diseases, migraine, disorders of carbohydrate metabolism, and mood disorders such as anxiety and depression.^55^^,^^56^ In addition, other causes of dizziness frequently overlap with MD during disease progression and should be promptly recognized and treated, including persistent postural-perceptual dizziness and BPPV.

Finally, a subset of patients with advanced bilateral MD may develop signs of bilateral vestibular hypofunction, requiring specific therapeutic approaches.

Recommendations for differential diagnoses

V Other episodic vestibular disorders (central and peripheral) should be considered in the differential diagnosis of MD. In urgent and emergency settings, clinical reasoning should follow evaluation protocols for acute vestibular syndromes. Vestibular migraine should be included in the differential diagnosis of MD, especially in the absence of the characteristic low-frequency hearing loss. (Strength of recommendation: strong; Level of evidence: moderate).
VI Comorbidities and conditions commonly associated with MD, such as migraine, autoimmune diseases, metabolic disorders, and mood disorders, should be investigated and appropriately managed. Detailed medical history and physical examination using the HINTS battery should be employed in the assessment of patients presenting with acute vertigo, especially in emergency settings, in patients presenting with acute vestibular syndrome and when performed by trained clinicians. (Strength of recommendation: strong; Level of evidence: high).
VII Disease progression should be monitored, considering the possibility of concurrent vestibular disorders, particularly persistent postural-perceptual dizziness, BPPV, and bilateral vestibular hypofunction. Comorbidities associated with MD, including migraine, metabolic disorders, mood disorders, and autoimmune diseases, should be investigated and treated to optimize disease control. (Strength of recommendation: moderate; Level of evidence: low).

## Objective

To review and provide evidence-based recommendations for the diagnosis and treatment of MD.

## Methods

A task force consisting of otolaryngologists and experts in otology and neurotology, including members and directors of the Brazilian Society of Otology (Sociedade Brasileira de Otologia, SBO) and the Brazilian Academy of Neurotology (Academia Brasileira de Otoneurologia, ABON), convened both in person and remotely to develop this guideline. Each participant was assigned responsibility for preparing an evidence-based section of the document, which was subsequently discussed collectively until consensus was achieved. A rapporteur drafted the final text, which was reviewed by coauthors and by the editor of the Brazilian Journal of Otorhinolaryngology-Head and Neck Surgery (BJORL-HNS).

This guideline does not replace individual clinical judgment. Physicians should always make decisions according to their training, scope of practice, and the best interests of their patients, even when such decisions differ from the recommendations presented herein.

For the critical appraisal and recommendation of therapeutic interventions, the grading system of the American College of Physicians (ACP) was adopted. Although the evaluation of diagnostic tests was also considered relevant, the ACP system is not ideally suited for this purpose. Therefore, the model proposed by the American Thyroid Association (ATA) was used, which considers criteria such as consecutive recruitment of patient’s representative of clinical practice, use of an appropriate reference gold standard, directness of the evidence, precision of diagnostic accuracy measures (including confidence intervals for sensitivity and specificity), and consistency of results among studies using the same test.

## Results

### Audiologic evaluation

#### Audiometry

Audiometry is the fundamental examination in the assessment of MD and constitutes one of its diagnostic cornerstones.^7^ The most important characteristic of hearing loss in MD is fluctuation during the early stages of the disease, which can be documented with serial audiometry. Audiometric testing is essential for differentiating probable from definite MD.

Initially, sensorineural hearing loss predominantly affects low frequencies, typically between 250 and 1000 Hz, distinguishing it from other causes of sensorineural hearing loss, such as presbycusis or noise-induced hearing loss, which preferentially affect high frequencies.^57^ As the disease progresses, there is a tendency for involvement of mid and high frequencies, resulting in a flatter or even downward sloping audiometric configuration.^58^

Hearing fluctuation represents the most characteristic and clinically relevant feature of hearing loss during the early stages of MD. This fluctuation may occur at different time points: during acute episodes of vertigo or aural fullness, between episodes, or over more prolonged periods. During an acute attack, temporary hearing deterioration is commonly observed, with partial or complete recovery after resolution of vestibular symptoms.^6^

Appropriate documentation of hearing fluctuation requires serial audiometry, preferably performed during different stages of the disease. Documented low-frequency hearing loss distinguishes probable from definite MD.^6^ The gradual deterioration process reflects progressive structural changes in the inner ear, including degeneration of hair cells and other cochlear structures.

Up to 50% of patients may develop bilateral MD, whereas 11%–23% present initially with bilateral symptoms. Because vertigo alone does not allow determination of the affected side, audiometry is the most useful examination for identifying laterality.^59^

Longitudinal studies have demonstrated that 82% of patients progress to moderate-to-severe hearing loss (mean >50 dB) over a 20-year period.^7^ This progression has direct implications for clinical management, influencing decisions regarding therapeutic interventions (conservative treatment, intratympanic procedures, or surgical approaches).^60^

Audiometry is also fundamental in the preoperative assessment of patients who are candidates for invasive or surgical procedures, such as intratympanic injections, Vestibular Neurectomy (VN), or labyrinthectomy. In these cases, documentation of residual auditory function is essential for determining the most appropriate surgical approach.^61^

A limitation of audiometry is the subjective nature of the test, which may be influenced by factors such as patient cooperation, the presence of intense tinnitus during testing, or attention disturbances related to vestibular symptoms.^62^

Audiometric findings should be interpreted in conjunction with other auditory and vestibular tests to provide a comprehensive assessment of MD. The combination of audiometry with objective measures, such as immittance testing, otoacoustic emissions, and auditory evoked potentials, may provide complementary information regarding middle and inner ear functional status.

Electrophysiologic testing aims to detect the presence of endolymphatic hydrops, a phenomenon associated with MD but not exclusive to this disease. These tests should be preferably performed close to periods of fluctuating symptoms, such as hearing loss, increased aural fullness, or tinnitus, thereby providing valuable support for diagnosis and longitudinal monitoring. During asymptomatic periods or between attacks, the diagnostic sensitivity of these tests may be reduced.

The most relevant examinations include otoacoustic emissions and Electrocochleography (ECochG).

### Otoacoustic emissions

The use of Distortion-Product Otoacoustic Emissions (DPOAEs) to detect endolymphatic hydrops is based on the hypothesis that not only the mechanical properties of the cochlea but also stereocilia position are altered in the presence of hydrops.^63^ Phase changes in low-frequency DPOAEs can be detected when the patient is moved from the upright to the supine position. Upon returning to the upright position, the phase returns to baseline within approximately 10 seconds. Although this is a rapid test, it still requires a tilt table.

Gerenton et al.^62^ reported 94% sensitivity and 88% specificity for the combination of DPOAEs with ECochG during the same session. Bachelet et al.^64^ demonstrated that when ECochG is combined with other functional tests, such as DPOAEs, diagnostic sensitivity increases significantly. In that study, the combination of functional explorations (ECochG and DPOAEs) with 3T MRI resulted in positivity rates of 97% for definite MD and 82% for probable MD.

### Electrocochleography

ECochG in MD is based on recording 2 principal components: the Summating Potential (SP) and the Action Potential (AP). The SP is generated predominantly by inner hair cells at the cochlear base in response to acoustic stimulation and reflects sustained depolarization of these cells during stimulus presentation. The AP represents the synchronized response of auditory nerve fibers to acoustic stimulation and corresponds to the distal portion of the cochlear nerve generating brainstem auditory evoked potentials.^65^

In endolymphatic hydrops, increased endolymphatic pressure causes displacement of the basilar membrane toward the scala tympani, resulting in increased/exaggerated depolarization of inner and outer hair cells. This alteration manifests as an increased SP, whereas the AP, which reflects nerve fiber activity, remains relatively unchanged. Consequently, the SP/AP ratio becomes elevated in patients with endolymphatic hydrops, constituting the main diagnostic marker of ECochG in MD.^66^^,^^67^

ECochG may be performed using different electrode placement methods, each with specific advantages and limitations. Although the transtympanic technique is considered the gold standard, it is invasive and requires placement of an electrode through the tympanic membrane onto the promontory. ECochG with 1000 Hz tone-burst stimuli requires the use of a transtympanic electrode.^67^

The use of a tympanic electrode resting on the tympanic membrane provides a favorable signal-to-noise ratio and good sensitivity for detecting cochlear potential abnormalities, while also being comfortable for the patient. For this method, established reference values for endolymphatic hydrops include an SP/AP amplitude ratio ≥35%, an SP/AP area ratio >1.66, and an AP latency difference between rarefaction and condensation clicks >0.35 ms. The combination of these 3 findings contributes to improved test sensitivity and specificity.^68^^,^^69^

To date, no reference values have been established for extratympanic electrodes (tiptrode or ear canal electrodes); therefore, their use is not recommended. Poor waveform morphology and difficulty interpreting low-quality ECochG recordings discourage clinicians from routinely using this tool.^7^

The clinical application of ECochG remains controversial because test sensitivity for MD ranges from 20% to 92%.^70^^,^^71^ ECochG using tympanic or transtympanic electrodes requires substantial technical expertise for electrode placement, test acquisition, and interpretation, demanding training and experience. Obeidat et al.^70^ evaluated 107 individuals with definite MD. Of 75 patients with unilateral MD, 72 (96%) had an abnormal SP/AP area ratio and 71 (95%) had an abnormal SP/AP amplitude ratio.

The considerable variability in results among different centers and operators may be attributed to differences in recording techniques, stimulation parameters, and interpretation criteria. This lack of standardization has limited both comparison across studies and broader clinical applicability of the examination.^69^

Combining ECochG with imaging and other functional tests may maximize diagnostic sensitivity and minimize the individual limitations of each method. Otoacoustic emissions, ECochG, and even MRI only detect endolymphatic hydrops, which is associated with MD but is not exclusive to the disease. Functional tests performed near the time of attacks may assist in diagnosis and clinical follow-up.

Recommendations for audiologic evaluation

VIII Serial audiometry is essential for the diagnosis and monitoring of MD, especially for documenting the characteristic hearing fluctuation observed during the early stages of the disease. (Strength of recommendation: strong; Level of evidence: high).
IX The combination of ECochG with otoacoustic emissions should be considered in cases of suspected MD to improve diagnostic sensitivity, particularly when performed during symptomatic episodes. (Strength of recommendation: moderate; Level of evidence: moderate).
X Pure-tone and speech audiometry combined with the glycerol test may be used to document hearing fluctuation and contribute to the diagnosis of MD. (Strength of recommendation: weak; Level of evidence: low).

## Vestibular testing

### Caloric test

Caloric testing is used to identify unilateral peripheral vestibular hypofunction and the stage of central compensation.^72^^,^^73^ It is currently believed that the impaired caloric response observed in patients with MD results from otolithic membrane herniation rather than from expansion of the endolymphatic space within the canal, as previously assumed.^74^

Caloric testing may demonstrate normoreflexia, hypo- or hyperreflexia, labyrinthine predominance, or directional preponderance. More recently, caloric testing has also been used to demonstrate activity in the affected ear through the observation of vestibular recruitment.^73^ This phenomenon occurs when the patient exhibits stronger responses to cold irrigation than to warm irrigation in the affected ear, indicating impaired peripheral vestibular function and hyperactivity of the contralateral vestibular nucleus.

### Video head impulse test

The Video Head Impulse Test (vHIT) is a vestibular assessment that uses high-frequency stimulation to evaluate the function of all 6 semicircular canals. In the early stages of MD, deterioration of the vertical semicircular canals tends to occur before involvement of the horizontal semicircular canal. Therefore, it is reasonable to hypothesize that vHIT may serve as a useful tool for differentiation in the early stages of the disease.^75^ Disagreement between vHIT and caloric testing (i.e., abnormal caloric responses in the presence of normal vHIT findings) has been considered a marker of MD.^76^^,^^77^

One possible explanation for this dissociation is the selective loss of type II hair cells in MD, leading to selective impairment of low-frequency labyrinthine sensitivity and abnormal caloric test results.^78^ More recently, however, this theory has been challenged. An alternative plausible explanation for such findings relates to the central mechanism of adaptation to MD-induced damage, which is effective only for physiologic high-frequency stimuli, but not for the non-physiologic low-frequency stimuli used during caloric testing.^79^ A third hypothesis proposes that impaired caloric responses in patients with MD result from otolithic membrane herniation, with consequent reduction of the post-caloric effect due to shielding and reduced stimulus efficacy.^74^

### Vestibular evoked myogenic potentials

Vestibular Evoked Myogenic Potentials (VEMPs) are sensitive to changes in intralabyrinthine fluid and pressure, as occurs in MD. VEMP responses vary according to disease stage and may show increased response amplitudes during attacks and reduced or absent responses in between attacks.^80^ These variations are related to the presence of endolymphatic hydrops in the saccule and/or utricle, which may represent increased sound transmission due to saccular occlusion (augmented response amplitudes) or, depending on disease stage and duration, varying degrees of sensory dysfunction involving both maculae (reduced amplitudes or absent responses).

Histopathologic studies suggest that the cochlea and saccule are the first inner ear structures affected by endolymphatic hydrops in MD, followed by the utricle and semicircular canals.^81^ Therefore, the use of VEMPs may allow early diagnosis, potentially even before the onset of hearing loss. In advanced cases, in which hearing loss is already established, VEMPs become particularly valuable because they do not depend on hearing acuity.^80^

In the functional vestibular evaluation of patients with MD, cervical VEMP (cVEMP) and ocular VEMP (oVEMP) show the highest prevalence of abnormalities, followed by caloric testing and then by vHIT. Based on histopathologic studies, this pattern of functional impairment correlates with the prevalence and progression of endolymphatic hydrops in the inner ear, reflecting greater involvement of the cochlea, saccule, utricle, and semicircular canals. The combination of VEMPs with other vestibular tests, such as vHIT and caloric testing, allows comprehensive mapping of the extent of functional impairment in the inner ear structures.^82^

Recommendations for vestibular testing

XI The dissociation between abnormal caloric testing and normal vHIT findings should be used as a complementary marker in the diagnostic evaluation of MD. (Strength of recommendation: moderate; Level of evidence: moderate).
XII VEMPs (cVEMP and oVEMP) should be included in the functional assessment of patients with MD to characterize the stage and extent of vestibular impairment. (Strength of recommendation: strong; Level of evidence: high).
XIII Vestibular recruitment observed during caloric testing may be considered an indicator of unilateral MD activity. (Strength of recommendation: weak; Level of evidence: low).

## Laboratory investigations

Laboratory tests are not direct diagnostic tools for MD, but they play an important complementary role in the clinical evaluation process.^83^ Their primary objectives are to exclude differential diagnoses or underlying conditions associated with endolymphatic hydrops that may exacerbate symptoms, and to identify possible secondary causes of hydrops, thereby assisting in treatment planning and prognostic assessment.^83^^,^^84^

Clinical conditions in which laboratory testing is particularly useful in the diagnostic investigation of MD include^7^^,^^83^, ^84^, ^85^:

Systemic inflammatory or infectious processes:

• Complete Blood Count (CBC).
• C-Reactive Protein (CRP).
• Erythrocyte Sedimentation Rate (ESR).

Metabolic dysfunctions:

• Disorders of carbohydrate metabolism: fasting glucose and insulin levels, glycated hemoglobin (HbA1c). In selected cases: glucose and insulin curves or oral glucose tolerance test with insulin measurements.
• Thyroid dysfunction: Thyroid-Stimulating Hormone (TSH) and Free Thyroxine (FT4).
• Dyslipidemia: lipid profile.
• Electrolyte disturbances: sodium, potassium, calcium, and magnesium

Specific infections: Syphilis: VDRL and FTA-ABS.

Autoimmune diseases:

• Systemic lupus erythematosus: Antinuclear Antibodies (ANA), anti-double-stranded DNA, anti-Sm, anti-Ro (SSA), anti-La (SSB).
• Sjögren syndrome: ANA, anti-Ro (SSA), anti-La (SSB).
• Systemic vasculitis: p-ANCA (MPO), c-ANCA (PR3).
• Autoimmune thyroiditis: anti-Thyroid Peroxidase (anti-TPO), anti-thyroglobulin.
• Antiphospholipid syndrome: lupus anticoagulant, anticardiolipin antibodies (IgM and IgG), anti-β2 glycoprotein I antibodies (anti-β2GPI; IgG and IgM).
• Autoimmune inner ear disease: anti-68 kDa antibodies (possibly directed against the HSP-70 antigen).

### Role of laboratory tests in phenotypic classification

In addition to their role in diagnostic investigation, laboratory tests also contribute to the phenotypic classification of MD.^85^ Immunologic testing may assist in identifying autoimmune etiologies, which carry prognostic implications and may predict responsiveness to immunosuppressive therapy.^86^, ^87^, ^88^

Genetic testing, such as Next-Generation Sequencing (NGS)-based gene panels, may identify: Mutations associated with the familial phenotype of MD^85^; Specific mutations in genes such as OTOG, COCH, and TECTA.^86^, ^87^, ^88^

### Cytokines and inflammation in Menière’s disease

Recent studies have investigated the role of proinflammatory cytokines in the pathophysiology of MD. Although still in the research phase, these findings may eventually enable patient stratification based on immunologic profiles and support the implementation of personalized biologic therapy.^86^, ^87^, ^88^

The following inflammatory profiles have been described:

• TNF-α: Elevated in subgroups with autoinflammatory activity. Associated with the genetic variant rs4947296 and activation of the NF-κB pathway.
• IL-1β: Elevated baseline levels in approximately 10%–20% of patients. This profile defines a phenotype associated with increased IL-6 expression and activation of the adaptive immune response.
• IL-6, CCL3, CCL18, and CXCL4: Additional inflammatory markers that may potentially assist in distinguishing clinical subgroups of the disease.

Recommendations for laboratory investigations

XIV A basic laboratory screening panel should be performed in all patients with suspected MD to exclude secondary causes and treatable comorbidities. Suggested tests include CBC, CRP, ESR, fasting glucose, HbA1c, 3- or 5-h glucose-insulin curve with 100 g of glucose, TSH and FT4, electrolytes (Na, K, Ca, Mg, Zn), lipid profile, serum cortisol, ferritin, vitamin B12, and VDRL/FTA-ABS (syphilis screening), selected according to clinical suspicion. (Strength of recommendation: moderate; Level of evidence: moderate).
XV Targeted immunologic/autoimmune investigation (ANA, anti-DNA, anti-Ro/La, anti-TPO, ANCA, antiphospholipid antibody panel, and, when available, auditory antibodies such as anti-HSP70/anti-68 kDa) should be ordered only when clinical findings or patient history are suggestive of systemic autoimmune disease, or when disease progression is atypical or refractory. (Strength of recommendation: weak; Level of evidence: low).
XVI Laboratory tests should not be used in isolation to establish the diagnosis of MD. Diagnostic evaluation should prioritize integration of medical history, serial audiometry, vestibular testing, and imaging, with laboratory investigations serving as a complementary tool for decision-making (etiologic assessment, differential diagnosis, and etiology-based therapeutic intervention). (Strength of recommendation: moderate; Level of evidence: low).

### Imaging studies

The traditional diagnosis of MD is based on clinical criteria, according to the American Academy of Otolaryngology-Head and Neck Surgery (AAO-HNS) guidelines, which, in their most recent version, do not recommend the routine use of imaging for diagnostic confirmation.^7^

However, advances in high-resolution neuroimaging, particularly gadolinium-enhanced MRI and high-definition CT, have expanded the role of imaging. These modalities now serve not only as tools for differential diagnosis but also as confirmatory, prognostic, and phenotypic stratification instruments.^14^

The historical development of MRI in MD may be divided into 3 phases:

1 Proof of concept (2005–2007): visualization of endolymphatic hydrops using contrast-enhanced imaging and 3D-FLAIR/3D-REAL sequences.^89^, ^90^, ^91^
2 Diagnostic optimization (2007–2020): standardization of imaging parameters, contrast administration routes, and endolymphatic hydrops grading methods.^92^, ^93^, ^94^, ^95^
3 Prognostic integration and personalized treatment (2020–present): use of imaging biomarkers, such as the Angular Trajectory of the Vestibular Aqueduct (ATVA), retrolabyrinthine bone thickness, and early detection of endolymphatic hydrops using high-resolution sequences, allowing personalized approaches according to disease endotypes.^96^, ^97^, ^98^, ^99^

In Brazil, adoption of these technologies should occur gradually, taking into account the availability of high-field MRI systems (≥3T), local expertise, and protocols adapted to regional realities. Standardization of imaging sequences, post-contrast timing, and interpretation criteria, aligned with international consensus recommendations, is essential to optimize clinical impact.^100^

### Visualization of endolymphatic hydrops

For many years, the feasibility of directly identifying endolymphatic hydrops in living patients, while clearly distinguishing endolymphatic from perilymphatic spaces, was questioned. This goal became achievable with advances in MRI technology.

Zou et al.^87^ demonstrated that gadolinium administered into the middle ear can permeate the round window membrane and selectively accumulate in the perilymph without diffusing into the endolymph. When administered intravenously, gadolinium enhances the Scala vestibuli and Scala tympani of the cochlea. Subsequently, Naganawa et al.^88^ showed that 3D-FLAIR sequences allow differentiation between enhancing perilymph and non-enhancing endolymph, with 4-hs identified as an optimal imaging delay.

In 2007, Nakashima et al.^91^ achieved the first direct in vivo visualization of endolymphatic hydrops in patients with MD using intratympanic gadolinium followed by a 3D-FLAIR MRI sequence after a 24-h delay. Since then, multiple studies have investigated different administration routes (intratympanic and intravenous) and new imaging sequences (3D-REAL IR), resulting in technical refinements and improved visualization of inner ear compartments.^92^, ^93^, ^94^^,^^96^, ^97^, ^98^, ^99^, ^100^

The degree of perilymphatic enhancement tends to be increased in patients with sensorineural hearing loss and more pronounced in MD-affected ears than in idiopathic hearing loss, possibly due to increased blood-perilymph barrier permeability.^101^ The combination of increased perilymphatic enhancement and the presence of endolymphatic hydrops may improve diagnostic sensitivity and specificity.^102^^,^^103^

### Beyond endolymphatic hydrops: biomarkers for endotype characterization

Visualization of endolymphatic hydrops plays a confirmatory role and increases diagnostic confidence; however, its isolated prognostic value remains limited. Correlation between imaging and histopathology has demonstrated the usefulness of the ATVA as a surrogate marker for the distal endolymphatic sac endotype: obtuse values are associated with a hypoplastic endotype (MD-hp), whereas more acute angles correspond to a degenerative endotype (MD-dg).^104^ A wider ATVA is linked to a thinner retrolabyrinthine bone, which can be readily measured on CT or MRI.^105^

### Clinical and prognostic applications

#### Confirmatory role

Multicenter studies indicate that gadolinium-enhanced MRI may be incorporated into diagnostic models with accuracy exceeding 90% (AUC = 0.907), especially in patients with incomplete or atypical symptoms.^106^ Kirbac et al.^104^ reported a sensitivity of 93.8% and specificity of 81.3% for MRI using intravenous gadolinium-based contrast agents.

#### Prognostic value and bilaterality

Imaging may identify endolymphatic hydrops in asymptomatic ears, suggesting an increased risk of bilateral disease. Patients with a hypoplastic vestibular aqueduct are at increased risk of bilateral involvement and require closer follow-up.^14^^,^^107^

#### Anatomic endotyping and treatment decision-making

The concept of clinical-radiologic endotypes ‒ MD-hp and MD-dg ‒ has therapeutic implications. MD-hp, likely congenital in origin, tends toward bilateral involvement, poorer prognosis, and greater surgical complexity, whereas MD-dg, likely acquired in origin (frequently associated with migraine), tends to remain unilateral and shows better response to Endolymphatic Sac Decompression (ESD).^14^^,^^108^, ^109^, ^110^

#### Complementary role of computed tomography

High-resolution CT evaluates vestibular aqueduct morphology, ATVA, and retrolabyrinthine bone thickness, and may serve as a surrogate marker for the vestibular aqueduct trajectory.^14^^,^^105^ These parameters contribute to phenotypic stratification and surgical decision-making, particularly when MRI is unavailable or contraindicated.

The evolution of imaging in MD has transformed the diagnostic paradigm, enabling more precise and personalized approaches. Gadolinium-enhanced MRI (preferably intravenous using a 3D-FLAIR protocol) should be employed selectively, strategically, and according to formal indications. CT represents a useful complementary tool for anatomic evaluation and phenotypic stratification. Clinical diagnosis remains central; however, imaging provides objective additional value, especially in uncertain, refractory, or surgical candidate cases. The progressive implementation of these technologies in Brazil should consider the realities of local health care facilities while striving for diagnostic excellence.

Clinical diagnosis should remain central, with imaging serving as a complementary tool in selected scenarios, such as diagnostic uncertainty, treatment planning, risk assessment of bilateral disease, and research settings. Standardization of imaging protocols ‒ including post-contrast timing and grading criteria for endolymphatic hydrops ‒ as well as adequate team training are essential to ensure reproducible results. Incorporation of these techniques into Brazilian institutions is recommended to occur gradually, according to equipment availability (particularly MRI systems ≥3T) and local expertise.

Where MRI is unavailable or contraindicated, high-resolution CT remains a valid tool for anatomic evaluation of the vestibular aqueduct and retrolabyrinthine bone and should be used whenever such information may influence clinical management. Furthermore, centers utilizing advanced imaging techniques are encouraged to participate in multicenter registries or collaborative protocols to generate prospective evidence on the impact of imaging biomarkers on clinical outcomes.

Recommendations for imaging studies

XVII In patients with unilateral symptoms and asymmetric hearing, gadolinium-enhanced MRI should be ordered to exclude vestibular schwannoma. (Strength of recommendation: strong; Level of evidence: moderate).
XVIII In cases with atypical symptoms or incomplete clinical diagnosis, gadolinium-enhanced MRI (3D-FLAIR/3D-REAL IR sequences) should be used for visualization of endolymphatic hydrops as a supportive imaging biomarker. (Strength of recommendation: strong; Level of evidence: high).
XIX Before invasive interventions (surgical planning or intralabyrinthine procedures), gadolinium-enhanced MRI should be performed to confirm laterality and exclude contralateral disorders that may alter management. (Strength of recommendation: strong; Level of evidence: moderate).
XX For monitoring the risk of bilateral disease, MRI combined with high-resolution CT should be considered to evaluate endolymphatic hydrops and vestibular aqueduct morphology in patients at increased risk (e.g., early contralateral abnormalities, family history, and prolonged disease course). (Strength of recommendation: moderate; Level of evidence: low).
XXI MRI + CT should be employed for phenotypic subtyping when anatomic endotyping may influence treatment decision-making (e.g., expected bilateral involvement or choice for surgery). (Strength of recommendation: moderate; Level of evidence: low).

## Dietary and lifestyle measures

Traditionally, nutritional management in MD has been based on restriction of salt, caffeine, and alcohol intake, founded on the hypothesis that these substances influence endolymphatic volume and, consequently, disease symptoms. However, recent evidence has challenged the efficacy of these conventional approaches and has pointed toward inflammatory mechanisms as more promising therapeutic targets.

The Cochrane systematic review conducted by Webster et al.,^108^ regarded as a gold standard in evidence-based medicine, found no Randomized Controlled Trials (RCTs) supporting the salt, caffeine, or alcohol restriction widely recommended in guidelines for MD, revealing a lack of robust evidence for such recommendations. This conclusion is reinforced by a Mendelian randomization study involving 941,280 participants, which identified no causal correlation between consumption of these substances and disease development, although potential effects in specific subgroups cannot be excluded.^108^ Mendelian randomization is particularly valuable because it uses genetic variants as instrumental variables, thereby minimizing confounding factors and reverse causality bias.

Nevertheless, more recent experimental studies have suggested a possible association between high salt intake and symptom worsening in individuals with MD, especially through inflammatory mechanisms. In an in vitro study, peripheral blood mononuclear cells from patients with MD exposed to high NaCl concentrations exhibited significantly increased expression of proinflammatory cytokines such as IL-1β and IL-6, as well as a specific IL-1β isoform associated with chronic inflammatory processes.^111^^,^^112^ These effects were not observed in healthy controls, suggesting an exaggerated immune response to sodium in susceptible individuals. It is important to emphasize, however, that these findings derived from an experimental study and still require confirmation in clinical studies.

The relevance of these findings is further strengthened by evidence supporting the key role of inflammation in MD pathogenesis. Frejo et al.^110^ identified 4 distinct immunophenotypes in patients with MD (allergic, autoimmune, autoinflammatory, and of low inflammatory activity), characterized by significant changes in cytokines such as IL-1β, TNF-α, and IgE. These findings reinforce the participation of systemic inflammatory mechanisms in disease pathophysiology, even during periods of clinical remission. In addition, Zhang et al.^111^ demonstrated, in an experimental model, a possible link between IL-1β-mediated inflammatory response and dysfunctional glutamatergic neurotransmission in MD, contributing particularly to symptoms such as hearing loss and vertigo.

These recent findings establish a direct connection between systemic inflammation, neuroinflammation, and the characteristic symptoms of the disease, providing a solid scientific basis for therapeutic approaches aimed at inflammatory modulation.

Based on this understanding, dietary interventions with antioxidant and immunomodulatory properties have been investigated as complementary therapeutic strategies. Di Paola et al.^112^ conducted a nonrandomized controlled trial in which supplementation with *Coriolus versicolor* (3 g/day for 6-months) was associated with significant improvement in clinical and biochemical parameters in patients with MD, including reduction in vertigo attack frequency, improvement in tinnitus perception measured with the Tinnitus Handicap Inventory (THI), improvement in emotional status measured with the Profile of Mood States (POMS), and normalization of biomarkers of oxidative stress and inflammation. The efficacy of *Coriolus versicolor* was attributed to activation of the Nrf2 antioxidant pathway and modulation of inflammatory proteins such as NF-κB, α–synuclein, GFAP, and Iba-1, in addition to restoration of mitochondrial function. It is important to note that this represents one of the first controlled trials investigating a specific anti-inflammatory dietary intervention in MD; however, the nonrandomized design limits the level of evidence.

Furthermore, correction of nutritional deficiencies, particularly vitamin D deficiency, may be relevant, as suggested by Bakhshaee et al.,^113^ who identified a significant association between low serum vitamin D levels and MD in observational studies. Based on a theoretical pathophysiologic model, Borghi and Pirodda^114^ proposed that systemic hemodynamic changes could contribute to the labyrinthine damage observed in MD. They suggested that omega-3 fatty acids, owing to their antioxidant, immunomodulatory, and vasoregulatory properties, warrant investigation as potential adjunctive agents in the management of this condition.

Although no clinical trials have yet been conducted directly involving patients with MD, the adoption of anti-inflammatory diets, such as the Mediterranean diet, is supported by recent meta-analyses demonstrating beneficial effects of this dietary pattern on inflammatory biomarkers (IL-6, TNF-α, and CRP) in different populations, albeit with variable magnitude and heterogeneity among studies.^115^, ^116^, ^117^, ^118^ These findings, along with the characterization of persistent inflammatory response in MD described by Frejo et al.,^110^ in which cytokines such as IL-6, TNF-α, IL-1β, and CRP are elevated in patient subgroups, provide a plausible pathophysiologic basis for considering dietary interventions with anti-inflammatory potential in disease management. However, the effectiveness of these interventions in patients with MD remains to be confirmed by prospective controlled clinical studies; at present, recommendations are based primarily on biological plausibility and indirect evidence.

Emerging evidence highlights the crucial role of water homeostasis modulation ‒ particularly through aquaporins ‒ in the pathophysiology of MD. In a controlled clinical study, Naganuma et al.^116^ demonstrated that an increase in water intake to 35 mL/kg/day significantly improved hearing ability and relieved vertigo in patients with MD after 2-years of follow-up. This finding is supported by the revised pathophysiologic model proposed by Takeda and Taguchi,^117^ who identified aquaporins as essential regulators of inner ear water homeostasis. The vasopressin-aquaporin system regulates endolymphatic fluid and electrolyte balance in a manner analogous to the renal system, and disturbances in this regulation may promote the development of endolymphatic hydrops. These findings suggest that adequate hydration may modulate aquaporin function and improve inner ear fluid balance. However, according to the most recent Cochrane review, the certainty of evidence supporting this recommendation is considered low.^111^

Beyond specific dietary interventions, recent evidence underscores the importance of lifestyle measures directed toward intestinal health. Mochizuki et al.^118^ identified a significant negative correlation between intestinal microbial diversity and MD duration, suggesting that dysbiosis may progress over time during the disease course. Although the exact mechanisms remain incompletely understood, alterations in gut microbiota may influence neuroinflammation and central immune regulation via the gut-brain axis.^119^, ^120^, ^121^, ^122^ Therefore, strategies aimed at restoring microbial diversity may be beneficial in patients with MD, although studies specifically targeting this population are still needed.

Lifestyle and dietary interventions for MD currently have very limited and low-certainty evidence of their efficacy.^111^^,^^123^ Although interventions such as abundant water intake or sleeping in darkness have been investigated, results remain uncertain, and robust clinical studies are lacking for the approaches most commonly recommended in clinical practice, such as salt and caffeine restriction or stress management.

Cordani and Briguglio^120^ suggested that a holistic approach involving healthy nutrition, physical activity, sleep hygiene, and stress management may benefit patients with MD. However, they acknowledged that these recommendations still lack support from high-quality randomized trials.

Finally, although pathophysiologic hypotheses involving the hypothalamic-pituitary-adrenal axis, vasopressin, and cortisol in endolymphatic volume regulation and neuroinflammation have been proposed, these mechanisms have not yet been validated as effective therapeutic targets in the clinical management of MD. Further studies specifically targeting this population are necessary to determine whether stress reduction and improved sleep quality truly provide objective clinical benefits.

In summary, there is biological plausibility and scientific rationale to support the adoption of the aforementioned different dietary and lifestyle strategies in the management of MD, including anti-inflammatory, antioxidant, water homeostasis-modulating, and gut microbiota-targeted interventions, as well as integrated health approaches. Nevertheless, the robustness of clinical evidence remains limited for most of these interventions. Therefore, prospective controlled studies specifically focused on patients with MD are recommended in order to strengthen the scientific basis and guide more precise recommendations for this population.

Recommendations for dietary and lifestyle measures

XXII Salt, caffeine, and alcohol restriction should not be recommended as specific therapeutic interventions for MD. (Strength of recommendation: moderate; Level of evidence: high).
XXIII Adequate water intake (approximately 35 mL/kg/day) may be considered a complementary strategy for endolymphatic homeostasis modulation. (Strength of recommendation: moderate; Level of evidence: moderate).
XXIV General lifestyle measures ‒ including sleep hygiene, stress management, regular physical activity, and a fractionated low-glycemic-index diet ‒ should be encouraged as part of a comprehensive management approach. (Strength of recommendation: weak; Level of evidence: low).

## Treatment of acute vertigo in menière’s disease

The treatment of acute vertigo in MD aims to provide rapid symptomatic relief during disabling attacks, which may last for several hours, while considering symptom severity, individual medication tolerance, and the risk of adverse effects.^6^ In general, treatment consists of a combination of vestibular suppressants (antihistamines, anticholinergics, and benzodiazepines) and antiemetics, administered in a stepwise manner at the lowest effective dose for the shortest possible duration to avoid impairment of vestibular compensation. In selected cases, depending on phenotype and clinical presentation, calcium channel blockers and corticosteroids may be considered, either systemically or via intratympanic administration.^124^

### Vestibular suppressants and antiemetics

These medications constitute first-line therapy during acute attacks and include 4 principal pharmacologic classes: antihistamines, anticholinergics, benzodiazepines, and antiemetics.^125^

Antihistamines

Antihistamines are widely used because of their favorable safety profile and efficacy. Common agents include:

- Meclizine: long duration of action (24-hs) and preferred during pregnancy. Usual dose: 25–100 mg/day.^126^
- Dimenhydrinate: effective via oral, intramuscular, or intravenous administration (50–100 mg, 3–4 times daily).
- Diphenhydramine: less commonly used but useful in acute settings.

Mechanism of action: Antihistamines exert anti-vertigo effects by modulating central and peripheral vestibular system activity through actions on histamine H1, H2, and H3 receptors. In the central vestibular system, these receptors are located in the vestibular nuclei, whereas in the peripheral system they are found in type II hair cells, calyceal afferent endings, and synaptic boutons. These agents reduce vestibular neuronal excitability, thereby alleviating vertigo and dizziness symptoms.

### Anticholinergics

Anticholinergics are indicated as an alternative to antihistamines. Examples include:

- Scopolamine: effective via transdermal or oral administration. It reduces the excitability of the vestibular nuclei. Usual dose: 10 mg orally every 8-hs or 1 transdermal patch every 72-hs.
- Atropine: currently less frequently used but may be administered in emergency settings. Dose: 0.4–0.6 mg intramuscularly or subcutaneously.

Mechanism of action: Anticholinergics act in the treatment of dizziness by blocking Muscarinic Acetylcholine Receptors (mAChRs) present in both the peripheral and central vestibular systems. Acetylcholine normally enhances vestibular pathway activity and sensitivity through rapid nicotinic receptor activation and slower muscarinic receptor modulation. In MD, hyperexcitability of these pathways contributes to symptoms such as vertigo and nausea. By antagonizing mAChRs, these medications reduce vestibular neuronal excitation, attenuate motion perception, and promote symptomatic relief.^125^

### Benzodiazepines

Benzodiazepines are prescribed in cases of failure of antihistamines or anticholinergics, or during more severe attacks. These include:

- Diazepam: 5–10 mg orally/day.
- Clonazepam: 0.25–0.5 mg orally, 2–3 times daily.
- Lorazepam and alprazolam may also be used.

These agents additionally exert anxiolytic effects, which may be beneficial in attacks with an important emotional component. They should be used cautiously because of the risk of sedation, dependence, amnesia, and, more rarely, respiratory depression (particularly with intravenous administration).

Mechanism of action: Benzodiazepines act on the GABAergic system as positive modulators of the GABA-A receptor, enhancing inhibitory neurotransmission in the Central Nervous System (CNS). This reduces the excitability of neurons causing vertigo.^127^^,^^128^

### Antiemetics

Antiemetics control severe nausea and vomiting, thereby improving patient comfort.^129^^,^^130^

- Ondansetron: 4 mg orally or intravenously, 2–3 times daily.
- Promethazine: 12.5–25 mg orally, intramuscularly, or rectally every 6–8 h.
- Metoclopramide: 20–30 mg/day orally, intravenously, or intramuscularly, divided into 2–3 doses. This medication should be used cautiously because of the risk of extrapyramidal side effects, including acute dystonia, marked motor restlessness, tremors, rigidity, and, in rarer cases of prolonged use, tardive dyskinesia characterized by repetitive involuntary movements, particularly involving the face and tongue.

Patients with persistent vomiting should also be assessed for dehydration and may require fluid replacement therapy.

Mechanism of action: Antiemetics act by blocking dopamine D2 receptors in the chemoreceptor trigger zone of the medulla oblongata in the CNS. This blockade inhibits stimuli causing nausea and vomiting, making these agents effective in controlling such symptoms during acute attacks.

### Considerations regarding prolonged use

Vestibular suppressants and antiemetics should not be used for prolonged periods after resolution of acute symptoms. Prolonged use may impair the process of central vestibular compensation, which is essential for long-term functional adaptation.^131^, ^132^, ^133^ In severe or refractory cases, medication intensification may be considered, always with careful assessment of the risk-benefit ratio.

### Calcium channel blockers

Calcium channel blockers are used primarily as maintenance therapy in recurrent vertigo but may also be employed during the acute phase of MD.^134^ Their indication may occur in different clinical settings: as adjuncts to first-line medications, in patients who continue to experience attacks despite prophylactic therapy; in the presence of contraindications to first-line agents; or when a vascular component is suspected to contribute to symptoms, such as in patients with the migraine-associated phenotype.^135^^,^^136^ In these situations, despite their delayed onset of action, calcium channel blockers may assist in modulating vestibular excitability and improving inner ear perfusion, thus contributing to symptom control. Major agents include:

- Flunarizine: 10 mg once daily. Long-term use may cause sedation and weight gain. There is also a risk of parkinsonism and depression.
- Cinnarizine: 25–75 mg/day. Similar to flunarizine but with a lower incidence of adverse effects.
- Nimodipine: used in some countries (e.g., the United States) at a dose of 30 mg twice daily.^137^
- Verapamil: also used off-label, generally at a dose of 120 mg every morning.^137^

Mechanism of action: These agents are believed to act as vestibular suppressants by blocking calcium channels present in vestibular hair cells, modulating their excitability. Studies in animal models of endolymphatic hydrops, as well as limited analyses of endolymph in patients with MD, indicate increased calcium levels, suggesting that disturbances in calcium homeostasis may contribute to vestibular symptoms. By inhibiting calcium influx, these agents may reduce vestibular hyperactivity, thus preventing symptoms such as vertigo and nausea. Flunarizine and cinnarizine also act on H1 and dopamine receptors, enhancing their anti-vertigo effects.^137^ In addition, these drugs reduce vascular tone, promote vasodilation, and improve tissue perfusion.^134^

### Corticosteroids

Although corticosteroids are more commonly used as maintenance or preventive therapy, they may also be indicated during acute episodes, especially in the presence of systemic autoimmune disorders, recurrent vertigo, or a sudden decrease in hearing thresholds.^138^ Common regimens include:

- Prednisone: 1 mg/kg orally once daily, up to a maximum dose of 60 mg.
- Dexamethasone: administered intratympanically to reach the inner ear, with or without concomitant intravenous administration.^139^^,^^140^

Mechanism of action: Corticosteroids are used in MD to alleviate vertigo and hearing loss, possibly by reducing inflammation and immune responses in the inner ear. The presence of glucocorticoid receptors in the inner ear suggests that these agents may exert a direct effect on intralabyrinthine fluid homeostasis.^140^ However, robust evidence of their efficacy is still lacking for both systemic and intratympanic administration, and results remain controversial because of variability in treatment protocols.^138^

### Anesthetics

In refractory situations, anesthetic agents with sedative properties may provide clinical benefit. Dowdy and Moore138 reported improvement in approximately 60% of patients with MD ‒ who had not responded to low-sodium diet, diuretics, and stress reduction ‒ after a single administration of droperidol or fentanyl. The effect was attributed to prolonged depression of the vestibular system.

Recommendations for pharmacologic therapies

XXV Vestibular suppressants (antihistamines, anticholinergics, and benzodiazepines) and antiemetics should be used as first-line treatment during acute attacks, administered in a stepwise manner for the shortest possible duration. (Strength of recommendation: strong; Level of evidence: moderate).
XXVI Prolonged use of vestibular suppressants and antiemetics after symptom resolution should be avoided because of the risk of delaying or impairing vestibular compensation. (Strength of recommendation: strong; Level of evidence: low).
XXVII Calcium channel blockers may be used during the acute phase in selected cases, especially in the presence of the migraine-associated phenotype, contraindications to first-line medications, or failure of initial therapy. (Strength of recommendation: moderate; Level of evidence: low).
XXVIII Systemic or intratympanic corticosteroids may be used during acute attacks associated with sudden hearing deterioration or suspected immune-mediated mechanisms, acknowledging that the available evidence is limited and heterogeneous. (Strength of recommendation: weak; Level of evidence: low).

## Vestibular rehabilitation

Postural balance control, gaze stability, spatial orientation, and navigation are highly integrative processes based on the interaction of different sensory modalities and internal spatial representation. Consequently, any damage to the vestibular system disrupts this integration.^141^^,^^142^ Vestibular Rehabilitation Therapy (VRT) has proven to be an effective method for controlling vestibular symptoms and has been applied to patients with various vestibular disorders.^143^

VRT includes adaptation, habituation, and substitution exercises that incorporate head and trunk movements to stimulate the vestibular system, improve the visual-vestibular interaction, and increase both static and dynamic postural stability. Therefore, these exercises can have a positive effect on quality of life by alleviating symptoms of dizziness and anxiety.^144^

The therapeutic approach to MD should prioritize conservative measures, particularly during the early stages of the disease and in patients with preserved hearing. The primary objective is to reduce the frequency and, secondarily, the severity of vertigo attacks, while minimizing hearing impairment, with the expectation that favorable control may also be associated with improvement in hearing and tinnitus. Conservative treatments are used regardless of hearing status, whereas destructive therapies are preferentially reserved for patients with established hearing loss. In bilateral cases, management becomes even more delicate because of the typically unilateral initial presentation and delayed contralateral involvement, further supporting a conservative therapeutic approach.^145^

VRT is considered a useful treatment option in MD, especially outside acute attacks, with a moderate level of evidence. Although a Cochrane review did not confirm a direct positive impact on balance and dizziness-related quality of life, the review included unilateral vestibular dysfunctions in general rather than MD specifically.^108^ Nevertheless, moderate to strong evidence supports VRT as a safe and effective therapeutic approach. When combined with psychotherapy ‒ particularly cognitive behavioral therapy ‒ vestibular rehabilitation has demonstrated significant benefits in dizziness-related symptoms, functional disability, and subjective distress.^144^^,^^145^

When conservative treatments (medications, lifestyle modifications, dietary interventions, and VRT) fail, intratympanic corticosteroids are recommended as second-line therapy. Third-line treatment depends on the patient’s auditory function and may involve endolymphatic sac surgery (when hearing preservation remains desirable) or intratympanic gentamicin therapy (associated with a higher risk of hearing loss). The final treatment option is destructive surgery (labyrinthectomy), which may be combined with Cochlear Implant (CI) or vestibular nerve section. After the aforementioned invasive procedures, VRT may also contribute to improving patients’ quality of life.^143^ Therefore, in many centers, VRT after destructive procedures is the accepted best practice for ensuring decreased postural sway and improved daily function for patients with advanced MD.^146^

In addition to vertigo attacks, patients with MD may also experience dizziness and imbalance. Vertigo attacks may lead to the loss of vestibular function, causing these chronic symptoms.^147^

Dizziness and imbalance not only impair patients’ quality of life but also increase their risk of falling. Studies have demonstrated that patients with unilateral vestibular hypofunction have a higher risk of falling than healthy individuals.^148^

VRT improves quality of life and reduces fall risk in patients with MD who experience balance instability and chronic dizziness. These patients become increasingly dependent on vision and proprioception to maintain balance. VRT can integrate visual, proprioceptive, and residual vestibular function, reduce the fear of vertigo episodes, improve patients’ confidence in maintaining balance, and reduce falls.^148^

Recommendations for vestibular rehabilitation

XXIX Vestibular rehabilitation should be used as an adjunctive conservative treatment for patients with unilateral or bilateral MD who present with chronic dizziness, postural instability, or persistent functional impairment, preferably outside acute attacks. (Strength of recommendation: strong; Level of evidence: moderate).
XXX Vestibular rehabilitation should be considered after ablative or destructive procedures (intratympanic gentamicin therapy, labyrinthectomy, or endolymphatic sac surgery) to optimize functional recovery, reduce postural instability, and accelerate vestibular adaptation. (Strength of recommendation: weak; Level of evidence: low).
XXXI Vestibular rehabilitation programs should be individualized according to disease stage, predominant phenotype (vertigo attacks, residual vestibular hypofunction, chronic imbalance), main functional limitations, and psychiatric comorbidities. Programs may include adaptation, habituation, and substitution exercises and, when indicated, integration with psychotherapy (particularly cognitive behavioral therapy). (Strength of recommendation: moderate; Level of evidence: moderate).

## Betahistine

Betahistine (N-alpha-methyl-2-pyridylethylamine) is one of the most commonly used medications worldwide for the treatment of MD. Its effects appear to involve both peripheral and central mechanisms of action, promoting vasodilation of the inner ear and acting on histaminergic receptors. Betahistine is indicated for MD and for the subacute treatment of dizziness in nonspecific labyrinthine disorders, functioning as a probable facilitator of vestibular compensation mechanisms.

### Mechanism of action

Although its exact mechanism of action has not yet been fully elucidated, betahistine is believed to exert peripheral effects by promoting vasodilation of inner ear vessels, as well as central effects by acting on the central histaminergic system as a weak H1 receptor agonist and H3 receptor antagonist.^149^, ^150^, ^151^, ^152^, ^153^

The central effects of betahistine appear to facilitate central vestibular compensation processes.^154^^,^^155^ Its peripheral effects, with improvement in the microcirculation of the cochleovestibular system, have supported its use in MD.^150^^,^^151^ The central histaminergic effects support its role as a vestibular facilitator, acting not as a vestibular sedative, but rather as a stimulator of vestibular compensation processes, potentially useful in various labyrinthine disorders.^152^^,^^153^^,^^155^

### Evidence of safety and efficacy

A favorable risk-benefit ratio has been demonstrated for the use of betahistine in the treatment of MD and vestibular symptoms associated with nonspecific labyrinthine disorders.^156^

A 2013 Cochrane systematic review suggested that, despite the low level of evidence available, patients with vestibular symptoms from different causes ‒ not only MD ‒ may benefit from betahistine through reduction of vestibular symptoms.^157^ The European guideline on MD management includes betahistine as a first-line treatment.^158^ Currently, betahistine is the most prescribed medication for MD in Europe.^159^^,^^160^

Definitive evidence supporting the efficacy of betahistine for MD control is still lacking. A multicenter, double-blind, randomized, placebo-controlled trial (BEMED trial) concluded that the effects of betahistine on vertigo control were not statistically superior to placebo.^161^ Three major Cochrane reviews addressing the use of betahistine in MD are available. The first review, published in 2001, concluded that there was insufficient evidence on any beneficial effect of betahistine in MD.^162^ The second review, published in 2016, evaluated the efficacy of betahistine for vertigo symptoms and concluded that low-quality evidence suggested that betahistine may have a positive effect in reducing vertigo symptoms.^157^ The third and most recent review, published in 2023, evaluated pharmacologic interventions for MD and concluded that available studies were not sufficiently robust to support definitive conclusions.^163^ Nevertheless, all 3 reviews concluded that betahistine has a high level of safety.

Two independent systematic reviews published in 2022 found that betahistine at different dosages (48 mg/day and 144 mg/day) probably does not reduce MD symptoms compared with placebo.^164^^,^^165^ Another review published in 2024 also identified heterogeneous efficacy results. Consistent with cluster analysis studies attempting to subdivide MD into clinical subgroups, these investigations suggest that future research based on immunologic subgroups may provide a more precise evaluation of the true efficacy of current treatments.^166^

### Dosage

The recommended dosage is 24–48 mg/day, divided into 2 or 3 daily doses. Exceptionally, dosages exceeding the conventional range, up to 144 mg/day, have been shown to be safe. More recently, extended-release formulations have become available and have demonstrated pharmacologic equivalence to conventional formulations while providing greater convenience in dosing schedules.

Recommendations for betahistine

XXXII Betahistine may be used as prophylactic therapy in MD with the aim of reducing recurrence and severity of vertigo symptoms, acknowledging that the evidence supporting efficacy is limited and heterogeneous. (Strength of recommendation: moderate; Level of evidence: low).
XXXIII Betahistine should generally be administered at conventional doses of 24–48 mg/day, although higher doses (up to 144 mg/day) may be considered in selected cases when well tolerated, despite no clear evidence of increased efficacy. (Strength of recommendation: moderate; Level of evidence: low).
XXXIV Extended-release betahistine formulations do not differ in efficacy or clinical indication from conventional formulations. (Strength of recommendation: weak; Level of evidence: low).

## Diuretics

Diuretics are widely used in the management of MD based on the assumption that reduction of extracellular fluid volume may decrease endolymphatic pressure and volume, either by increasing drainage of endolymph or by reducing its production in the stria vascularis.^138^ Pharmacologically, diuretics act by inhibiting sodium reabsorption at different segments of the nephron, thereby increasing urinary excretion of sodium and water.^138^ According to their mechanism of action, these agents are classified as thiazide diuretics (inhibitors of sodium and chloride reabsorption from the distal convoluted tubules), potassium-sparing diuretics (inhibitors of sodium-potassium exchange within collecting ducts), loop diuretics (inhibitors of sodium reabsorption in the loop of Henle), and carbonic anhydrase inhibitors (which increase sodium, potassium, bicarbonate, and water excretion).^167^

Historically, thiazide diuretics, with or without potassium-sparing agents (such as hydrochlorothiazide/triamterene or spironolactone), have been the most frequently prescribed medications for MD, followed by the carbonic anhydrase inhibitor acetazolamide as second-line therapy.^7^ However, their use requires caution: thiazide diuretics are contraindicated in patients with gout, and potassium-sparing diuretics are contraindicated in patients with acute or severe renal failure. Monitoring of blood pressure and serum electrolytes is therefore essential.^7^

Despite the theoretical rationale supporting their use, the clinical efficacy of diuretics in the treatment of MD remains uncertain. Systematic reviews indicate that most available studies are of low methodological quality, with small sample sizes and inconsistent results. In their systematic review, Crowson et al.^165^ analyzed 19 studies, most of which provided low-level evidence, and suggested that oral diuretic therapy may reduce the frequency of vertigo episodes, although hearing benefits were less consistent. Similar findings were reported by Rosenbaum et al.,^166^ who identified only 2 randomized trials with small sample sizes and very low-certainty evidence. Nevertheless, the authors noted that diuretics have an acceptable safety profile.

A Cochrane review identified only 2 additional randomized trials meeting the inclusion criteria: one evaluating isosorbide and another evaluating a combination diuretic therapy. However, the certainty of evidence was considered very low, precluding robust conclusions regarding the efficacy of these interventions.^163^ Glycerol, an osmotic diuretic, has produced a transient hearing improvement in patients with suspected MD and is therefore occasionally used as a diagnostic test.^163^

In the 2017 international consensus on treatment of MD, diuretics were recommended as first-line medical treatment despite the fragility of the available evidence. Hydrochlorothiazide, acetazolamide, and chlorthalidone were considered the most frequently used agents, in decreasing order, and were recommended for the potential reduction of vertigo episode frequency (level of evidence: 4; strength of recommendation: C), provided contraindications and adverse effects are appropriately considered.^145^

In summary, although widely prescribed and associated with a relatively favorable safety profile, diuretics still lack robust evidence of their efficacy in controlling MD symptoms.^170^

Recommendations for diuretics

XXXV Oral diuretics may be considered an option for reducing the frequency of vertigo episodes in patients with MD when conservative measures (guidance on sleep, hydration, diet, and symptomatic treatment) are insufficient. (Strength of recommendation: weak; Level of evidence: moderate).
XXXVI Diuretics should not be prescribed with the sole purpose of improving hearing or tinnitus. Hearing benefits are inconsistent and unproven. (Strength of recommendation: weak; Level of evidence: low).
XXXVII Diuretics should be prescribed on an individual basis with active monitoring (blood pressure, serum electrolytes, renal function, blood glucose, and drug-drug interactions); their use should be avoided in high-risk situations (severe renal failure, uncorrected hypo- or hyperkalemia, active gout, and severe hyponatremia depending on the type of diuretic). (Strength of recommendation: weak; Level of evidence: low).

## Other pharmacologic measures

MD has a variable and often unpredictable clinical course. While many patients respond well to first-line measures, such as dietary modification, diuretics, and betahistine, a subset of cases remains symptomatic, with recurrent vertigo episodes or progressive hearing loss.^145^ Consequently, several medications have been used for different therapeutic purposes, including modulation of inflammatory response, reduction of vestibular excitability, suppression of acute attacks, and even preservation of auditory function. Although not all of these options are supported by high-level evidence, many have already become part of clinical practice in specialized centers based on preliminary data or off-label use.

Systemic corticosteroids, particularly oral corticosteroids, may play a relevant role in the management of MD in selected situations, such as cases of sensorineural hearing loss, especially when there is strong suspicion of an immune-mediated phenotype. Despite the limited clinical evidence of their efficacy in classic MD, corticosteroid use is supported by pathophysiologic rationale and clinical observations in selected settings.^168^, ^169^, ^170^, ^171^ However, a 2023 Cochrane systematic review identified only 1 randomized trial evaluating oral corticosteroid therapy in MD, which demonstrated low methodological quality and inconclusive results regarding the efficacy of corticosteroids in reducing vertigo episodes.^172^

Although several corticosteroids may be used in clinical practice, the most common regimen consists of prednisone at a dose of 1 mg/kg/day for 7–14 days, followed by gradual tapering.^145^ During treatment, close clinical follow-up and laboratory monitoring are recommended, including assessment of blood glucose, lipid profile, and bone mineral density, given the potential systemic adverse effects of corticosteroids.

Intratympanic corticosteroid injection has become established as an effective rescue therapy in refractory MD, particularly when conventional medical treatment fails or when systemic corticosteroid use is contraindicated. This route allows local administration of dexamethasone or methylprednisolone directly into the middle ear, achieving high inner ear concentrations through diffusion across the round window membrane while minimizing systemic effects. Recent studies have demonstrated significant improvement in vertigo frequency, with hearing preservation and a low incidence of adverse effects. The most commonly used protocol consists of weekly dexamethasone injections (4–24 mg/mL) for 2- to 4-weeks, with repeat injections according to clinical response.^173^

Intratympanic gentamicin may be used for vertigo control in refractory MD prior to more invasive surgical procedures. However, according to a Cochrane review, the available evidence is of very low quality because of the limited number of studies and imprecise results.^172^ Despite reported benefits in some cases, the effects on hearing are uncertain, and treatment should therefore be reserved for selected patients under strict audiologic monitoring. Both therapies will be discussed in detail later.

Benzodiazepines may be used as adjunctive therapy in MD, particularly during episodes of acute vertigo. They act as central vestibular suppressants, reducing the excitability of the vestibular nuclei by enhancing inhibitory GABAergic neurotransmission. Although clinically effective for attack control, benzodiazepines do not modify the natural history of the disease and should be used cautiously, preferably for short periods, because of the risk of tolerance, dependence, and interference with central vestibular compensation. Their use is most appropriate during phases of acute vestibular decompensation or in patients with anxiety-related comorbidities, always under medical supervision.^174^

Antidepressants may play a complementary role in the treatment of patients with associated emotional comorbidities, such as anxiety, depression, insomnia, or other disorders that may amplify the perception of vestibular symptoms. Commonly used agents include selective serotonin reuptake inhibitors, such as sertraline,^175^ and serotonin-norepinephrine reuptake inhibitors, such as venlafaxine.^176^ In some cases, particularly those with overlap with vestibular migraine, amitriptyline may also be considered.^177^

Calcium channel blockers represent an alternative therapeutic approach in the management of MD, especially in patients with overlap with vestibular migraine or vasomotor disorders. Agents such as flunarizine, cinnarizine, and nimodipine have been investigated because of their vasoregulatory and neuronal excitability-stabilizing properties, which contribute to reducing the frequency and severity of vertigo attacks and, in some cases, improving auditory symptoms.^178^ Although some evidence suggests potential clinical benefits, the quality of available studies is limited, and adverse effects, such as sedation, weight gain, and extrapyramidal symptoms (particularly with flunarizine), require caution.^179^ Therefore, their use should be reserved for selected cases after failure of conventional therapies and should be accompanied by careful medical evaluation and regular monitoring for adverse effects.

The use of anticonvulsants, such as topiramate, may be considered in selected cases of MD with the migraine-associated phenotype, i.e., when there is overlap with typical vestibular migraine symptoms such as photophobia, phonophobia, nausea, or a history of migraine.^180^

Although MD has well-established therapeutic approaches, including low-sodium diet, betahistine, and diuretics, a significant proportion of patients may remain symptomatic or develop further hearing loss. In such cases, complementary pharmacologic strategies become essential and should be tailored to the predominant clinical phenotype, associated comorbidities, and previous response to first-line therapies. Treatment decision-making should therefore balance risks and benefits, prioritizing control of disabling symptoms while minimizing functional impairment.

Recommendations for other pharmacologic measures

XXXVIII Systemic corticosteroids should not be routinely used in classic MD; their use should be considered only in selected cases, such as sudden hearing loss or suspected immune-mediated phenotype. (Strength of recommendation: weak; Level of evidence: low).
XXXIX Complementary pharmacologic therapies (benzodiazepines, antidepressants, calcium channel blockers, or anticonvulsants) should be used only in selected cases, according to the predominant clinical phenotype, associated comorbidities, and persistence of symptoms despite first-line treatment. (Strength of recommendation: weak; Level of evidence: low).

## Intratympanic corticosteroid therapy

Intratympanic injection has become established as an effective route of administration in the management of vertigo in MD, with increasing popularity over the past 2 decades.^172^ Among the agents used, intratympanic corticosteroids ‒ particularly dexamethasone and methylprednisolone ‒ represent a safe alternative to ablative therapies, allowing symptom control while preserving hearing.^181^^,^^182^ Their effects appear to result from immunomodulation, reduced inflammatory processes, and improved endolymphatic homeostasis.^183^^,^^184^

Recent systematic reviews reinforce the role of intratympanic corticosteroids in the management of MD. Webster et al.,^169^ in a Cochrane review including 10 randomized trials (n = 952), demonstrated that intratympanic dexamethasone may provide temporary improvement in vertigo, although without significant effects on hearing or tinnitus. However, an increased risk of tympanic membrane perforation was observed. In a similar vein, Wu et al.,^178^ in a meta-analysis of 12 studies, demonstrated the superiority of gentamicin over corticosteroids in vertigo control at 6-months, although this difference diminished after 1-year. Corticosteroids, in contrast, were superior in hearing preservation, consolidating their role as an alternative when hearing preservation is a priority. Lee et al.,^179^ in a meta-analysis of 5 studies, compared dexamethasone vs. methylprednisolone and suggested slight superiority of methylprednisolone in vertigo control, although both agents had similar efficacy in hearing preservation.

Two network meta-analyses further refined comparisons among different treatment protocols. Hao et al.,^182^ including 10 studies, and Cao et al.,^181^ including 9 randomized trials, confirmed that gentamicin is more effective in vertigo control, albeit at the cost of a higher risk of hearing loss, whereas corticosteroids offer a more favorable auditory safety profile.

Devantier et al.,^180^ in a systematic review of 3 randomized trials, reported marked heterogeneity among protocols, with overall modest and temporary benefit. These findings reinforce the need for multicenter clinical trials using standardized protocols to strengthen the evidence base.

Martínez et al.^183^ demonstrated significant improvement in vertigo and quality of life without hearing deterioration after dexamethasone treatment. Beyea et al.,^184^ in a prospective review of 106 patients, also observed sustained vertigo reduction over 1-year, with hearing preservation.

Another study evaluating 30 patients with refractory MD, who did not respond to standard medical therapy, used intratympanic dexamethasone injections.^185^, ^186^, ^187^, ^188^ The mean Dizziness Handicap Inventory (DHI) score decreased from 91.58 (80–100) to 31.00 after 3-months (p = 0.00). At subsequent follow-up evaluations, mean DHI scores were 51.50, 46.6, and 50.90 at 6-, 12-, and 24-months, respectively (p = 0.04; 0.35; 0.49). At the end of 24-months, 23.8% of patients were free of vertigo (p = 0.01). No hearing improvement >10 dB was observed during follow-up, and 6.6% of patients experienced hearing deterioration. No major complications were reported.

Regarding methylprednisolone, Patel et al.^186^ conducted a randomized double-blind trial involving 60 patients with MD. Patients were assigned to treatment with either methylprednisolone or gentamicin. In the intention-to-treat analysis, the mean number of vertigo attacks in the final 6-months decreased from 19.9 to 2.5 in the gentamicin group (87% reduction) and from 16.4 to 1.6 in the methylprednisolone group (90% reduction). The mean difference between groups was -0.9 (95% CI: -3.4 to 1.6). Non-responders received additional injections, with 2 patients switching from methylprednisolone to gentamicin. Both medications were well tolerated, with 6 adverse events reported (3 in each group), the most common being minor ear infections.^189^, ^190^, ^191^, ^192^, ^193^, ^194^, ^195^, ^196^ However, She et al.^197^ followed 17 patients with intractable MD treated with intratympanic methylprednisolone and observed disease control rates of 94% and 81% at 6- and 24-months, respectively.

Finally, novel sustained-release formulations such as OTO-104 (dexamethasone gel) have been investigated. Phase II multicenter trials showed benefit in reducing vertigo frequency, with patients requiring only a single injection for clinical benefit lasting up to 3-months. However, phase III results were heterogeneous, and some studies failed to replicate these benefits.^185^^,^^191^ These findings reinforce the potential of innovative therapies but remain insufficient to support commercial recommendation, highlighting the need for additional studies.

Recommendations for intratympanic corticosteroid therapy

XL Intratympanic corticosteroids (dexamethasone or methylprednisolone) should be used as rescue therapy for vertigo control in patients with MD refractory to conventional medical treatment, particularly when hearing preservation is a priority. (Strength of recommendation: strong; Level of evidence: high).
XLI Intratympanic gentamicin should be used for vertigo control in selected cases of refractory MD when symptom control is the primary goal and the risk of hearing loss is considered acceptable, especially in patients with limited functional hearing. Detailed counseling and strict audiologic monitoring are required before treatment. (Strength of recommendation: strong; Level of evidence: high).
XLII Methylprednisolone should preferably be used in patients with refractory MD, based on evidence suggesting similar or slightly superior efficacy in vertigo control. (Strength of recommendation: moderate; Level of evidence: high).

## Intratympanic gentamicin therapy

Over recent decades, intratympanic drug administration has become established as an alternative approach in the management of refractory MD. Among the available options, corticosteroids and gentamicin are the principal agents used. Technically, medication is instilled into the middle ear through the tympanic membrane, either by direct injection or via a ventilation tube, and reaches the inner ear via absorption through the round window membrane.

Dose regimens and frequency of administration vary substantially in the literature, ranging from weekly serial application protocols to spaced and response-titrated strategies.^192^^,^^193^ This variability reflects the absence of a universally accepted regimen and, consequently, the lack of a true gold standard treatment for the disease.

From a pathophysiologic perspective, aminoglycoside ototoxicity has been recognized since the mid-20th century and results primarily from damage to cochlear and vestibular hair cells. In MD, this property is therapeutically exploited with the aim of reducing vestibular function in the affected ear. Thus, without vestibular function in the inner ear, the frequency of attacks may be reduced, and central compensation facilitated, provided that the contralateral ear maintains adequate function.

Although all aminoglycosides may affect hearing, gentamicin has a relatively greater vestibulotoxic than cochleotoxic profile, which has historically favored its use for this indication.^194^^,^^195^ Nevertheless, irreversible hearing loss remains a real possibility, with apparently higher risk in high-dose or multiple-daily regimens. In addition, individual factors, including genetic susceptibility, may increase vulnerability.^172^^,^^196^ Consequently, low-dose regimens with titration according to clinical response and serial audiometric monitoring have gained increasing acceptance.

Stokroos and Kingma,^194^ Postema et al.,^195^ Bremer et al.,^196^ Shamas et al.,^197^ and Choudhary et al.^198^ evaluated adult patients with definite unilateral MD according to the 1995 AAO-HNS criteria or equivalent definitions, all refractory to medical therapy. Comparators were placebo or no intervention. In one of the trials, intratympanic corticosteroid therapy was assessed in an additional study arm (analyzed separately in a parallel review). A common feature of these studies was marked heterogeneity regarding dose, volume, and number of injections, limiting direct comparisons and robust meta-analyses. Vertigo was assessed as a dichotomous outcome (improvement vs. no improvement) in some studies and as a continuous outcome using subjective severity scores (0–3) or annual frequency of attacks in other studies. Hearing was assessed by Pure-Tone Audiometry (PTA) ‒ mean thresholds at 0.5, 1, 2, and 4 kHz ‒ in most trials, although lack of dispersion measures in some studies precluded quantitative synthesis at the review level.

Stokroos and Kingma^194^ administered gentamicin at 30 mg/mL in a volume of 4 mL, with repeat injections every 6 weeks until either clinical response or predefined discontinuation criteria were reached. Over follow-up ranging from 6- to 28-months, 11 of 12 patients in the gentamicin group reported improvement (ranging from “significant reduction” to “no complaints”), compared with 2 of 10 in the placebo group. Annual attack frequency decreased from approximately 12 to 2 episodes/year among treated patients. Hearing deterioration was variable and, on average, did not differ significantly from controls, although individual cases of clinically relevant decline were documented.

Postema et al.^195^ conducted a randomized, double-blind, placebo-controlled trial in which patients initially underwent ventilation tube placement. Participants received either gentamicin (30 mg/mL, 0.4 mL, once weekly for 4-weeks) or normal saline. Vertigo was assessed using a 4-point scale (0 = none; 3 = severe). After 12-months, mean vertigo scores decreased from 2.1 to 0.5 in the gentamicin group, compared with 2.0–1.8 in the placebo group. In addition, 9 of 16 treated patients were asymptomatic at study completion, whereas none of the 12 controls achieved this outcome. Hearing loss, expressed as the extended Fletcher index, increased by a mean of 8.1 dB in the gentamicin group and remained unchanged in the placebo group. Aural fullness improved significantly in treated patients, whereas no relevant differences were observed for tinnitus.

Bremer et al.^196^ conducted a double-blind, 3-arm randomized trial in which all participants received 4 injections, administered weekly: 4 injections of placebo; 2 injections of gentamicin (40 mg/mL) alternating with 2 injections of placebo (low-dose group); or 4 injections of gentamicin (high-dose group). The study was designed for 24 months of follow-up but was terminated prematurely after interim analysis because of concerns regarding hearing deterioration. The DHI was used as the primary outcome measure. Reduction in DHI scores was more pronounced in the high-dose group (median reduction: 41-points) than in the placebo group (20-point reduction) and the low-dose group (10-point reduction). However, mean PTA loss was 27.4 dB in the high-dose group, compared with 0.9 dB in the low-dose group and 10 dB in the placebo group. Three participants exceeded the predefined threshold for significant hearing loss (>30 dB), 2 of whom had received gentamicin. Interestingly, the low-dose group showed less hearing loss than the placebo group, which the authors attributed to the natural course of MD. Also, 1 patient in the placebo group developed significant hearing deterioration (>30 dB), attributed to the disease itself rather than treatment.

Shamas et al.^197^ published a prospective comparative study, described as case-control, including 60 patients with refractory MD randomly assigned to 3 groups: gentamicin (40 mg/mL, 2 mL), dexamethasone (4 mg/mL, 0.5 mL), and placebo (normal saline). Follow-up lasted 6 months, focusing on hearing and tinnitus outcomes. Audiometric evaluation and the THI were applied. The 2023 Cochrane review considered only the gentamicin vs. placebo comparison, highlighting the benefit of controlling tinnitus at the expense of increased risk of hearing deterioration.^108^ Vestibular symptom data, however, were not reported.

Choudhary et al.^198^ randomized 32 patients to low-dose gentamicin (20 mg/mL, 1–3 applications according to symptoms) or conservative management, with 6 months of follow-up. Vertigo control, assessed according to the 1995 AAO-HNS vertigo control classification (classes A to F according to the proportion of attacks after treatment, with A = Complete control and F = Worsening), was substantial: 87.5% achieved class A and 12.5% class B among treated patients. Further measurable hearing loss occurred in 12.5% of cases, without statistical significance compared with controls. Despite the prospective design and favorable clinical outcomes, the lack of blinding and small sample size limited the results.

The Cochrane reviewers emphasized the heterogeneity of application protocols, with variability in concentrations, volumes, and number of doses, preventing standardization of a single therapeutic regimen. They also highlighted diversity in outcome measurement: some studies used the 1995 AAO-HNS vertigo control classification, whereas others relied on subjective scores or annual attack frequency; only 1 trial evaluated quality of life using the DHI. Regarding hearing outcomes, only part of the studies provided sufficient PTA data. Overall, the Cochrane review concluded that intratympanic gentamicin probably improves vertigo control compared with placebo or no treatment, but it may be associated with hearing deterioration, particularly in higher-dose protocols. Certainty of evidence was rated as low to very low because of the small number of participants, methodological heterogeneity, and incomplete reporting of adverse events.^108^

In this context, it is important to broaden the perspective beyond Cochrane data by incorporating additional comparative synthesis studies. The meta-analysis by Hao et al.^182^ introduced indirect comparison with intratympanic corticosteroids, while the systematic review by Devantier et al.^180^ updated the analysis relative to placebo. These studies provide additional perspectives relevant to clinical judgment and the formulation of recommendations at the task force level.

Hao et al.^182^ analyzed 10 RCTs (n = 455) and found that both intratympanic gentamicin and corticosteroids were superior to placebo in vertigo control. Indirect comparison between gentamicin and corticosteroids revealed no significant difference in anti-vertigo efficacy. However, corticosteroids were superior in hearing preservation, with a mean PTA difference of approximately 6.5 dB and 7%–8% gain in speech discrimination.

More recently, Devantier et al.^180^ conducted a systematic review of RCTs comparing gentamicin with placebo or no treatment (3 studies; n = 65) and concluded that gentamicin reduces the frequency and severity of vertigo attacks, although overall certainty of evidence is very low because of small sample sizes, protocol heterogeneity, and risk of bias.

In summary, analyzing the Cochrane systematic review as well as the individual studies that comprise this review and more recent investigations, the body of evidence consistently points in the same direction: intratympanic gentamicin probably reduces vertigo attacks in the short and medium term when compared with placebo or no intervention. However, the magnitude and durability of benefit vary among studies, partly because of heterogeneity in treatment regimens and outcome measures. Conversely, the risk of hearing deterioration is real and appears to increase with cumulative exposure, although MD itself may also contribute to progressive hearing loss. Specifically, findings from Bremer et al.^199^ suggest that higher-dose regimens may produce more pronounced reductions in DHI scores, albeit at the cost of more substantial hearing deterioration.

Adoption of low-dose protocols with titration according to clinical response, with discontinuation once satisfactory vertigo control is achieved or clinically significant hearing deterioration occurs, appears to be a prudent strategy balancing efficacy and safety. It should also be noted that in situations where hearing preservation is a priority (e.g., only-hearing ear), intratympanic corticosteroids represent an attractive alternative. Corticosteroids have anti-vertigo efficacy that can achieve good results, while not posing a significant hearing risk.^183^^,^^185^

In clinical practice, intratympanic gentamicin is recommended for carefully selected patients with unilateral MD, disabling vertigo, and failure of medical treatment. Patients must be clearly informed of the potential for irreversible hearing loss, and monitoring should include not only vertigo symptoms but also hearing status. Baseline audiometry is recommended, as well as serial audiologic monitoring using standardized assessment tools for vertigo progression (1995 AAO-HNS vertigo control classification and/or DHI).

The literature still lacks standardized protocols, which should explicitly define concentration, volume, interval, and criteria for repeat injections or discontinuation. Adverse events should also be reported according to CONSORT-Harms guidelines. Important gaps remain larger trials, standardized outcomes, follow-up beyond 24-months, and direct comparisons with corticosteroids are research priorities.

Recommendations for intratympanic gentamicin therapy

XLIII Intratympanic gentamicin may be used for vertigo control in patients with definite unilateral MD refractory to medical treatment, provided that the contralateral ear maintains adequate function. (Strength of recommendation: moderate; Level of evidence: moderate)
XLIV Gentamicin should be administered using low-dose or response-titrated protocols, with treatment discontinuation once satisfactory vertigo control is achieved or any clinically significant hearing deterioration occurs. (Strength of recommendation: weak; Level of evidence: low).
XLV In patients for whom hearing preservation is a priority (e.g., only-hearing ear or high auditory demands), intratympanic corticosteroids should be considered as the initial alternative to gentamicin because of their superior auditory safety profile. (Strength of recommendation: moderate; Level of evidence: moderate).

## Endolymphatic sac decompression

Endolymphatic sac surgery occupies an important historical role in neurotology, particularly in the management of MD refractory to medical treatment. The hypothesis that MD is related to excessive accumulation of endolymph in the membranous labyrinth (endolymphatic hydrops) led, in the early 20th century, to attempts to surgically treat the condition through drainage or decompression of the endolymphatic sac. In 1927, Georges Portmann performed the first experimental surgery by creating a fistula between the endolymphatic sac and the dura mater in an attempt to drain excess endolymph.^199^ In subsequent decades, several variations of the technique emerged, including simple sac decompression, sac opening with shunt, and the use of synthetic materials such as Silastic.^200^, ^201^, ^202^, ^203^ In the 1970s and 1980s, RCTs began to question the efficacy of surgery, demonstrating that part of the observed benefit might be attributable to placebo effects or to the natural history of the disease itself.^204^^,^^205^ Nevertheless, endolymphatic sac surgery has remained an option for hearing preservation in patients who do not respond to medical treatment and in whom more ablative procedures, such as labyrinthectomy or VN, should be avoided.

The classic indications for ESD include patients with a diagnosis of MD who experience recurrent, disabling vertigo attacks refractory to appropriate clinical management, such as low-sodium diet, diuretics, corticosteroids, and betahistine.^206^^,^^207^ It is also considered an option in cases where preservation of residual hearing is desired, in contrast to ablative techniques. Patients with bilateral vestibular dysfunction or those at high risk for more invasive intracranial procedures may also benefit from a less aggressive technique, although its true clinical benefit remains debated.

The traditional ESD surgical technique involves several well-established steps. The procedure is performed under general anesthesia, typically through a retroauricular approach. Initially, the surgeon performs a retroauricular incision with muscle plane dissection to expose the mastoid cortex. This is followed by a simple mastoidectomy with identification of key anatomic landmarks, including the sigmoid sinus, posterior fossa dura, and posterior semicircular canal. After identifying the region of the endolymphatic sac adjacent to the posterior fossa dura, the surgeon carefully performs bony decompression by removing the bone overlying the sac and exposing its external wall. In some variations, only bony decompression is performed, whereas in others the sac wall is incised, with or without insertion of material to maintain drainage, such as Silastic fragments.^208^

Potential ESD complications, although relatively uncommon, include both general otologic surgical risks and procedure-specific risks. These include dural injury with cerebrospinal fluid leak, sigmoid sinus injury, meningitis, sensorineural hearing loss resulting from surgical manipulation or the natural course of disease, and facial paralysis.^209^ The rate of major complications is relatively low, which contributes to the procedure still being considered a viable option in the therapeutic armamentarium.

ESD has been investigated for several decades as a surgical intervention intended to preserve hearing in patients with MD refractory to medical treatment. The first randomized double-blind trial evaluating the role of interventions at the level of the endolymphatic sac was published by Thomsen et al.^201^ in 1981. In this study, 30 patients with refractory MD were randomized to undergo endolymphatic sac shunt surgery or mastoidectomy alone (placebo procedure), with blinding of both patients and evaluators and standardized 12-month follow-up. Outcomes included frequency and severity of vertigo attacks, auditory symptoms (hearing loss and tinnitus), and quality-of-life measures, with daily recording of vestibular episodes. The results showed significant improvement in vestibular symptoms in both groups, without statistically significant differences between the shunt and placebo groups, suggesting that the observed effects could largely be explained by nonspecific responses to the surgical setting. Hearing outcomes also showed no consistent differences between groups, and there was no evidence of a specific hearing benefit from the active procedure. Major limitations of this study included the small sample size (n = 30), which reduced statistical power to detect moderate differences; surgical techniques and outcome criteria that would now be considered outdated; and the possibility of crossover effects or natural disease variability. Because of its randomized double-blind design, the study represents level 1b evidence according to the Oxford Centre for Evidence-Based Medicine (OCEBM), and its predominant interpretation has supported a recommendation against widespread adoption of the procedure as clearly superior to placebo in terms of vertigo control.

The same authors subsequently published a 3-year follow-up extension of the same cohort, detailing medium-term clinical outcomes after active vs. placebo procedures. The extended follow-up sought to determine whether differences would emerge over a longer time horizon, given that the natural history of MD includes intermittent fluctuations and spontaneous remission. After 3-years, the clinical improvement observed during the first year persisted in many patients; however, there continued to be no statistically significant difference between the shunt group and the simple mastoidectomy group, reinforcing the hypothesis of a surgical placebo effect or nonspecific benefit of the surgical intervention itself. The authors argued that the natural history of the disease ‒ with periods of remission ‒ and the impact of prior medical treatment precluded direct causal attribution to surgery. Regarding limitations, in addition to the previously mentioned limited sample size, the study-maintained outcome criteria and measurements that were not harmonized with later consensus standards (e.g., AAO-HNS classification), and there was potential for missing data and follow-up bias. In terms of evidence level, the follow-up study remains within the category of RCTs (1b) and reinforces a cautious or negative recommendation regarding the specific benefit of shunt surgery compared with sham mastoidectomy.^210^

In contrast to the RCTs, over subsequent decades numerous case series and prospective cohort studies have advocated the clinical efficacy of ESD in controlling vertigo attacks. One of the most extensive reports was the case series published by Huang^200^ (addressed in reviews and compilations), in which experience with thousands of procedures was presented to support the effectiveness of the technique as a hearing-preserving intervention. In this large-volume series, reported vertigo control rates frequently approached or exceeded 70%–80% in the long term, and many authors recommended ESD as an option when conservative measures failed. Case series generally describe variable techniques (simple decompression, decompression with Silastic, shunting), with heterogeneity in indication criteria, outcome measures, and follow-up duration. Limitations inherent in case series include the lack of a control group, selection bias (patients selected for surgery), reporting bias, and preferential publication of positive outcomes. Furthermore, technical variability and lack of standardization in measurement tools hinder comparisons and quantitative synthesis. Therefore, although these cases series suggest consistent benefit for many patients, they constitute lower levels of evidence (generally levels 2–4 depending on study design) and provide recommendation grades ranging from B to C when considered in isolation.^203^

Paparella and Goycoolea^208^ reported their experience with one of the largest case series of the time, including more than 170 individuals. The authors proposed that the procedure is intended to enhance absorption of endolymph in the sac, potentially reducing endolymphatic pressure and, consequently, clinical symptoms. Results indicated significant vertigo control in a large proportion of patients, with substantial to complete improvement in approximately two-thirds to three-quarters of the sample. Hearing preservation was also observed in a relevant proportion of cases, while major complications were rare, reinforcing the safety profile of the surgery. However, the study had important limitations, including the lack of a control group, sample heterogeneity, and lack of standardized criteria for outcome assessment, limiting comparisons with other treatment modalities or with the natural course of the disease.

Paparella and Sajjadi^209^ further explored the topic in a study dedicated to the diagnostic and therapeutic rationale of the technique. Approximately 100 patients were evaluated, with particular emphasis on detailed description of surgical indication criteria, technical principles, and clinical outcome analysis. Once again, findings showed satisfactory vertigo control in most cases and low associated morbidity, supporting the characterization of the procedure as posing lower risk than destructive techniques such as VN. The authors emphasized that surgery should be reserved only for patient’s refractory to medical treatment, reinforcing its selective application. Nevertheless, study limitations included its observational nature, absence of randomization, and methodological difficulties in outcome assessment, such as variability in audiometric testing and lack of long-term follow-up for all patients.

In 1988, Paparella and Sajjadi^210^ specifically addressed recurrence after initial surgery by analyzing 26 patients who underwent revision surgery. The study demonstrated that the most common causes of failure were related to anatomic or cicatricial changes, such as extrasaccular fibrosis, osteoneogenesis, and aditus block syndrome, which compromised endolymphatic sac function. Revision surgery restored symptom control in a substantial proportion of cases and provided additional insight into underlying pathophysiologic mechanisms. Despite its clinical relevance, the study had limitations, including the small sample size, selection bias ‒ since only patients with recurrence were analyzed ‒ and lack of standardized postoperative audiologic evaluation.

A 1994 review published by Paparella and Sajjadi^211^ compiled 2 decades of accumulated surgical experience. The authors reported vertigo improvement rates reaching up to 90%, depending on the definition of control and follow-up duration. They also emphasized the safety of the procedure, underscoring that major complications were rare, and that cochlear function was preserved in many patients. The contribution of this study lies in its case series design, with detailed technical descriptions and discussion of surgical variations, which helped establish the procedure as routine practice in some otologic centers. However, limitations included the nonrandomized nature of the evidence, absence of comparison with alternative procedures, and difficulties inherent in long-term follow-up.

Subsequently, Sajjadi et al.^212^ specifically investigated outcomes in older patients, a population of growing interest because of increased longevity. Among 27 patients who responded to follow-up questionnaires, 23 reported significant improvement in vertigo and 19 stated that their hearing ability had either improved or was maintained at preoperative levels. The absence of major complications reinforced the safety of the procedure in this age group. However, the external validity of the findings was limited by the small sample size and use of retrospective questionnaires, which are subject to recall and response bias, as well as by the lack of a comparison group.

Finally, Paparella^213^ published a review focused on revision endolymphatic sac surgery, integrating clinical data and surgical observations. The author highlighted that failure of the initial surgery was frequently associated with scarring, anatomic abnormalities, or obstructions preventing adequate sac function. When properly performed, reintervention was able to restore symptom control in a substantial proportion of patients, supporting the hypothesis that MD has a strong component of endolymphatic malabsorption. As a limitation, the study remained descriptive in nature, without prospective design or control group, relying on accumulated clinical observations. Nevertheless, its relevance lies in its contribution to understanding disease pathophysiology and justifying further interventions.

Seeking prospective data with standardized patient-oriented outcome assessment, Durland et al.^214^ published a prospective study in 2005 evaluating symptom-specific and general health outcomes after ESD. The investigators incorporated validated patient-reported outcome instruments and focused on vestibular measures (frequency and severity of vertigo episodes), quality-of-life parameters, and auditory function. The findings showed statistically significant improvement in vestibular symptom scores and quality-of-life measures after surgery, supporting the position that, in selected settings and with appropriate technique, ESD may provide measurable clinical benefit. However, the study acknowledged heterogeneity in hearing outcomes ‒ some patients experienced postoperative hearing deterioration based on changes in PTA thresholds ‒ and noted that the magnitude of vestibular benefit varied across subgroups. Limitations included the lack of randomization and a control group, potential observer bias, and inability to distinguish specific procedural effects from nonspecific effects related to surgical care and intensive follow-up. In terms of evidence level, it corresponds to a prospective observational study with level 2–3 evidence (depending on detailed design) and, in isolation, provides a moderate recommendation grade (B) when combined with other observational evidence.

Given the controversy between the results of RCTs and case series, systematic reviews and meta-analyses have attempted to synthesize evidence and assess the certainty of effects. A 2015 systematic review by Lim et al.^203^ examined the literature published between 1990 and 2014, including RCTs, controlled studies, and case series, applying critical appraisal tools such as GRADE and AMSTAR. The review showed that although many studies reported vertigo control in a substantial proportion of patients undergoing ESD, overall methodological quality was low to moderate, with considerable risk of bias and heterogeneity in outcomes and techniques. The authors concluded that no robust new evidence contradicted the findings of Thomsen’s double-blind trial or unequivocally demonstrated a specific benefit of ESD beyond placebo effect, and that the certainty of estimates was frequently low. As limitations of the review itself, the authors highlighted the scarcity of contemporary RCTs and dependence on variable-quality case series data. Therefore, the review suggested a cautious recommendation (grade C) for ESD as an option, emphasizing the need for additional RCTs using standardized criteria.^215^, ^216^, ^217^

A review published in 2017 by Flores García et al.^218^ provided a broad synthesis of indications, surgical techniques (simple decompression vs. shunting with or without Silastic), outcomes, and complications of ESD. The authors reviewed both historical and contemporary literature and emphasized that findings remain contradictory: while many case series report satisfactory vertigo control, the double-blind trial and systematic reviews continue to question whether the benefit is procedure specific. The review also highlighted technical aspects that may influence outcomes ‒ such as sac opening, use of Silastic material, and extent of mastoidectomy ‒ and discussed evidence suggesting that the use of Silastic may not provide additional benefit and may even be associated with poorer hearing outcomes in some series. Limitations included the lack of standardization in definitions of success, variability in follow-up, and absence of standardized assessment of functional hearing outcomes such as speech discrimination. Thus, the review recommended that ESD indications should be carefully considered, preferably in experienced centers, and that patients should be informed of uncertainties regarding specific benefits and potential hearing risks. From the standpoint of evidence level, the review reinforced the perception that high-quality direct evidence is scarce and that recommendations remain moderate to weak in strength.

More recent meta-analyses using modern criteria have attempted to quantify the combined effects of ESD on vertigo and hearing. The meta-analysis by Szott et al.^206^ applied PRISMA/Cochrane methodology, aggregated data from comparative studies and case series, and analyzed functional outcomes, vertigo control, and auditory measures (PTA and speech discrimination). The results showed that, in terms of functional level and vertigo control, ESD produced favorable effects, with many patients achieving clinically meaningful reduction in vestibular episode frequency. However, there was measurable negative impact on hearing outcomes, including increased mean PTA thresholds and significant reduction in speech discrimination scores of clinically relevant magnitudes. The authors argued that although vestibular improvement appears consistent, the potential cost in terms of auditory function makes careful risk-benefit assessment essential for decision-making. Limitations of the meta-analysis included heterogeneity among studies (surgical technique, inclusion criteria, follow-up duration), possible publication bias, and reliance on aggregated rather than individual patient data for more refined analyses. In terms of evidence level, the meta-analysis provided stronger evidence (1a), although the final recommendation was interpreted as moderate (B) given the mixed profile of benefits and risks.

More recent comparative studies have attempted to contrast decompression techniques with alternative approaches, including Endolymphatic Duct Blockage (EDB), to determine whether other strategies may provide superior efficacy or auditory safety profile. A multicenter study and comparative analyses retrospectively evaluated cohorts undergoing ESD vs. EDB or other technical variations.^219^ Some studies suggested that EDB techniques may provide comparable vertigo control with potential differences in hearing outcomes, although results were inconsistent across centers. Major limitations included the observational design, selection of patients for specific techniques (indication bias), and technical variability limiting generalizability. Regarding evidence level, these observational comparisons remain level 2–3 evidence, providing conditional support (Grade B–C) for considering alternative approaches depending on specific treatment goals (e.g., hearing preservation vs. vestibular control).

Studies and case series published from 2020 to 2023 continued to report ESD outcomes with emphasis on patient-centered outcomes and objective hearing assessment. Contemporary prospective studies have emphasized the use of validated MD questionnaires, functional level scales, and standardized PTA and speech discrimination measurements, allowing more robust analysis of vertigo control and hearing preservation. For example, 2022–2023 studies comparing simple ESD, ESD with Silastic implantation, and alternative techniques found that simple sac decompression without Silastic tended to produce better hearing outcomes in some series, whereas insertion of Silastic material did not appear to add benefit and was occasionally associated with poorer outcomes. These recent contributions reinforce the need for technical standardization and better-designed prospective randomized studies to distinguish specific technical effects from contextual effects. Persistent limitations remain: few contemporary RCTs, sample sizes often insufficient for subgroup analyses, and residual heterogeneity in diagnostic and inclusion criteria. In terms of aggregated evidence, these observational and comparative studies provide additional indirect support (level 2–3 evidence) and imply a conditional recommendation (B–C) for ESD in selected centers with informed patient consent.^220^

When considering “all published studies” on ESD, it is important to note that the currently available literature combines different types of evidence: (1) 1 classic RCT and its follow-up questioning specific benefit; (2) Numerous case series and large prospective cohort studies, some reporting favorable vertigo control outcomes; (3) Systematic reviews and meta-analyses synthesizing these data and critically evaluating certainty of evidence; and 4) Contemporary comparative observational studies contrasting techniques and reporting differences between vestibular control and auditory impact.^204^^,^^205^

Recent literature suggests that ESD may provide good vertigo control for many patients, although evidence remains heterogeneous. The only available double-blind RCT did not show superiority of shunting over sham mastoidectomy, raising the possibility of placebo effect, although the study has important methodological limitations. Current meta-analyses indicate functional improvement and vertigo reduction but also report risk of hearing loss and a decline in speech discrimination in a proportion of patients. Overall quality of evidence ranges from low to moderate. Therefore, judicious use of the technique is recommended, with detailed discussion with patients regarding uncertainties and risks, preferably in experienced centers. To decisively strengthen the level of recommendation, contemporary multicenter RCTs with robust sample sizes, blinding whenever feasible, and standardized outcomes would be required.

Recommendations for endolymphatic sac decompression

XLVI ESD is indicated for patients with definite MD who experience recurrent, disabling vertigo attacks refractory to medical treatment, particularly when hearing preservation and avoidance of ablative procedures are desired. (Strength of recommendation: moderate; Level of evidence: moderate).
XLVII ESD reduces the frequency and severity of vertigo attacks in a substantial proportion of patients, although there is no robust evidence of unequivocal superiority over surgical placebo effects. (Strength of recommendation: weak; Level of evidence: high).

## Vestibular neurectomy

In cases refractory to intensive medical treatment, surgical intervention becomes an essential option to relieve disabling vertigo.^221^^,^^222^ Among destructive surgical options, VN is widely accepted as an effective method for controlling peripheral vertigo, particularly because of its ability to preserve auditory function.^221^, ^222^, ^223^

The concept of sectioning the auditory nerve to treat vertigo dates back to Charcot in 1874. In the early 20th century, Walter Dandy popularized VN at Johns Hopkins Hospital, performing 692 procedures between 1924 and 1946. Initially, Dandy performed complete sectioning of the eighth cranial nerve, but he later modified the technique to a partial VN that preserved hearing, substantially expanding surgical eligibility.^224^ His procedures had a remarkable mortality rate of less than 1% for the time (2 of 692 patients, due to meningitis). However, Dandy’s expansion of indications to include “pseudo-MD” and bilateral cases, without strict diagnostic criteria, later drew criticism and led to the perception that many of his patients would likely be considered misdiagnosed by current criteria.^225^

### Efficacy of vestibular neurectomy

Ablation of peripheral vestibular function is recognized as the most successful method to relieve vertigo caused by disorders of the vestibular labyrinth and/or vestibular nerve.^223^ VN has demonstrated high success rates for vertigo control, with complete relief in 97.8% of patients in a series of 45 cases.^223^ Another retrospective study of 44 patients undergoing retrosigmoid VN for disabling MD unresponsive to medical treatment showed that vertigo resolved in 77.3% of patients and improved in 13.6%, resulting in significant improvement in 90.9%.^221^ Similar results were observed in a series of 102 patients, in which complete vertigo control reached 100% 2-years after surgery.^226^

The efficacy of VN is based on the ability of the peripheral and central vestibular systems to adapt to unilateral vestibular loss through neural plasticity and substitution by other sensory inputs to restore balance.^223^ In many patients, quality of life improved significantly after VN, with most patients moving from moderate or severe disability to either being symptom-free or having only mild symptoms.^221^^,^^227^ Patients with “otolithic catastrophes of Tumarkin” (drop attacks), a severe symptom of MD, also showed elimination of these episodes in 11 of 12 cases after VN.^221^

Studies of post-VN vestibular function indicate that neurectomy may provide complete vestibular denervation. Tests such as caloric testing, vibration-induced nystagmus, vHIT, and VEMPs have shown absent or abnormal responses in most operated patients, confirming complete ablation of vestibular function.^228^^,^^229^

### Risks and complications of vestibular neurectomy

Although effective, VN is associated with significant risks and complications. A common complication of Middle Fossa VN (MFVN) is transient facial nerve paresis, observed in 15 (33%) of 45 patients in one study, with recovery within 1–4-months.^223^ Other serious complications include:

• Meningitis: reported in 1 patient.^223^
• Cerebrospinal fluid leak: 1 patient required lumbar drainage.^223^ In another series, 1 patient required reoperation for repair.^221^^,^^227^
• Epidural hematoma: reported in 1 patient, requiring surgical exploration.^223^
• Prolonged ataxia or imbalance: more patients experienced prolonged ataxia after VN (5 patients) than after labyrinthectomy (2 patients). Some VN patients (all but one aged <60 years) had prolonged imbalance lasting from 6-months to 3-years.^223^

Length of hospital stay and length of disability before return to work are also considerably longer with VN. In one study, the mean hospital stay was 6-days and return to work occurred after 5.9-weeks for VN, compared with 2.5-days and 3.6-weeks, respectively, for labyrinthectomy.^223^

MFVN may require 3–4 h of operative time. However, more recent approaches, such as the retrosigmoid and presigmoid retrolabyrinthine approaches, have shorter operative time (<2-hs in some retrosigmoid cases) and lower rates of major complications.^221^^,^^227^

Despite efforts to preserve hearing, some patients undergoing MFVN may experience postoperative hearing loss. In one series, 7 of 39 patients had significant hearing loss, attributed to injury to the cochlear nerve by traction on the inferior vestibular division.^223^ Development of contralateral MD was also observed in 2 patients after VN in one series.^223^

### Comparison with labyrinthectomy and other therapies

VN and labyrinthectomy are both considered effective ablative procedures for vertigo control in unilateral MD.^223^ However, important differences influence the choice of procedure:

• Hearing preservation: selective VN is the procedure of choice when hearing preservation is indicated and feasible. In one study, hearing was preserved in 82% (32 of 39) of patients undergoing selective VN.^223^ In contrast, labyrinthectomy is a destructive procedure that results in permanent hearing loss in the affected ear and is generally indicated for patients with severely impaired or no hearing.^223^
• Recovery time and cost: labyrinthectomy is associated with faster recovery and lower cost. Mean hospital stay and time to return to work are approximately twice as long with VN than with labyrinthectomy.^223^ The total cost of VN (surgical, anesthesia, and hospital fees) is more than twice that of labyrinthectomy.^223^
• Complications: VN is associated with a greater number and severity of complications, including facial paresis, meningitis, cerebrospinal fluid leak, and epidural hematoma. In one study, labyrinthectomy was complicated by postoperative hyponatremia in 1 patient.^223^
• Vestibular asymmetry: laboratory evidence suggests that vestibular asymmetry after labyrinthectomy is less pronounced than that after VN, which may be associated with more rapid and complete balance recovery.^223^

VN also compares favorably with other approaches, such as ESD. A comparative study of 30 patients with intractable MD (21 ESD, 9VN) showed that VN achieved 100% vertigo control, whereas ESD achieved 52.4% control and 19.0% improvement. However, ESD showed potential to improve hearing and tinnitus, which was not observed with VN.^230^

Other ablative therapies include intratympanic gentamicin injections. Although intratympanic gentamicin is an effective option, it poses the risk of hearing loss (approximately 26%) and more intense tinnitus.^221^^,^^227^ Some studies have reported that VN has a higher vertigo control rate and lower risk of hearing loss than intratympanic gentamicin.^227^^,^^231^

VN is indicated for patients with unilateral MD, disabling vertigo unresponsive to conservative treatment (for at least 6-months), good general health, adequate central compensatory capacity, and willingness to accept the potential risks associated with the procedure.^222^^,^^223^

Different surgical approaches include MFVN, retrosigmoid VN, and presigmoid retrolabyrinthine VN.^221^^,^^223^^,^^226^^,^^227^ The retrosigmoid approach is often preferred because of its safety and ease of access, allowing a more complete transection of the vestibular fibers.^226^ The presigmoid retrolabyrinthine approach, particularly with endoscopic assistance, provides a wide visualization of the cerebellopontine angle, minimizes cerebellar retraction and the risk of facial nerve injury, ensures a precise result, and preserves the cochlear fibers.^227^ Accurate identification of vestibular nerve fibers and their separation from cochlear and facial nerve fibers is essential for surgical success and complication avoidance. Intraoperative neurophysiologic monitoring is critical for selective identification of cranial nerves and verification of complete vestibular nerve sectioning.^222^^,^^227^

Functional recovery after VN may require substantial adaptation. Although acute vertigo is relieved, patients may experience residual balance dysfunction, such as gait deviation, particularly under low-vision conditions and during rapid head movements, underscoring the importance of visual input in vestibular compensation.^232^ Persistent dizziness or recurrent vertigo in some cases may be attributed to incomplete vestibular nerve sectioning, nerve regeneration, or the presence of vestibulocochlear nerve anastomoses.^222^^,^^229^

In summary, VN is a highly effective surgical intervention for controlling disabling vertigo in patients with refractory MD, especially those with preserved auditory function.^223^^,^^227^ While offering excellent vertigo control and the advantage of hearing preservation in most cases, VN is associated with a higher risk of serious complications and a longer, more costly recovery period compared with labyrinthectomy.^223^ The choice of surgical approach should be individualized, considering the patient’s profile, hearing level, general health, and willingness to accept the risks. Interdisciplinary collaboration between neurotologists and neurosurgeons is crucial for optimizing outcomes.^226^^,^^227^

The available evidence, primarily from retrospective and prospective cohort studies and case series, consistently demonstrates that VN provides superior vertigo control and long-term improvement in quality of life for carefully selected patients. Although it is an invasive procedure with inherent risks, its high success rate for vertigo control and potential for hearing preservation make it a valuable treatment option when conservative options have failed, and maintenance of hearing is clinically desirable. There is moderate evidence of high VN efficacy, even compared with intratympanic gentamicin, in patients with intractable vertigo and useful hearing. Because VN does not completely compromise auditory function, it may replace surgical labyrinthectomy in selected cases.^227^

Recommendations for vestibular neurectomy

XLVIII VN should be considered in patients with definite unilateral MD and disabling vertigo refractory to medical treatment for at least 6-months, particularly when functional hearing is present in the affected ear and central compensatory capacity is adequate. (Strength of recommendation: moderate; Level of evidence: low).
XLIX VN is one of the most effective ablative procedures for vertigo control in refractory MD, with high rates of complete or significant vertigo relief (approximately 90%–100%), including cases of Tumarkin otolithic crisis. (Strength of recommendation: moderate; Level of evidence: low).
L Compared with surgical labyrinthectomy and intratympanic gentamicin, VN may be preferred when hearing preservation is desirable and feasible, as it provides superior vertigo control with a greater likelihood of maintaining functional hearing, despite higher surgical morbidity. (Strength of recommendation: moderate; Level of evidence: low).
LI VN should be performed only in specialized centers, by teams experienced in skull base surgery, using intraoperative neurophysiologic monitoring, and after detailed patient counseling regarding potential risks (facial paresis, cerebrospinal fluid leak, meningitis, prolonged imbalance) and the longer functional recovery period. (Strength of recommendation: weak; Level of evidence: low).

## Labyrinthectomy

Labyrinthectomy is an inner ear ablative procedure indicated to completely eliminate peripheral vestibular function on the affected side and thereby achieve control of disabling dizziness refractory to less invasive therapeutic approaches. It is avoided as a first-line option because, by definition, it results in profound hearing loss when performed as a transmastoid labyrinthectomy, the classic technique, or when used as part of approaches to the lateral skull base. Thus, it is an excellent surgical treatment option for patients affected by disorders that have already completely compromised residual hearing, for whom it represents a gold-standard option for vertigo control.^7^^,^^233^

### Indications and patient selection

Main indications

• Symptomatic unilateral MD with disabling vertigo despite appropriate diet, diuretics, vestibular rehabilitation, and less invasive procedures, such as intratympanic gentamicin or corticosteroid injections, or ESD.^7^^,^^233^
• Level of hearing not suitable for rehabilitation with conventional hearing aids.^7^
• Drop attacks (Tumarkin crisis) that are very frequent and/or disabling, with failure of conservative therapies to achieve adequate control.^7^

Critical considerations in patient selection

• Risk of progression to bilateral disease manifestations, either at the time of evaluation or later in the disease course. This should be exhaustively discussed with the patient before proceeding with any labyrinthine ablation.^234^
• If hearing rehabilitation is needed, associated strategies should be considered, such as simultaneous or staged labyrinthectomy with CI in an ear with no useful hearing.^233^^,^^235^
• In bilateral disease or when laterality is uncertain, nonablative alternatives or selective VN should be carefully weighed, preferably in centers experienced with these approaches, to ensure better patient outcomes.^233^

### Labyrinthectomy techniques

#### Transmastoid labyrinthectomy

This technique uses the standard retroauricular approach typical of microscopic ear surgery, followed by cortical mastoidectomy with careful and accurate identification of the posterior, superior, and lateral semicircular canals. From that point, systematic removal of the endosteum and extirpation of the vestibular apparatus (semicircular canals and vestibule) are performed. As in other ear surgeries, particular caution is recommended to avoid injury to the facial nerve, sigmoid sinus, and tegmen tympani. Complete extirpation is essential for sustained vertigo control; for this reason, this is considered a radical ablative technique.^236^

Its advantages include the extradural nature of the approach, which implies a shorter operative time compared with other approaches, as well as a favorable and slightly shorter learning curve. Nevertheless, appropriate indication and execution of the procedure are associated with consistently high vertigo control rates.^237^

#### Labyrinthectomy and cochlear implant

This is not exactly an alternative technique, but rather the possibility of combining procedures in either a single surgical stage or 2 separate stages. This combination is indicated when the goal is both to eliminate vertigo and to control MD symptoms, while also rehabilitating hearing in the same ear. It may be performed simultaneously (single stage) or sequentially in 2 stages. Studies have demonstrated elimination or at least significant control of vertigo in most cases, in addition to significant hearing gains after CI.^236^

#### Translabyrinthine approach in specific settings

The translabyrinthine approach is also frequently referenced in other surgeries, such as treatment of vestibular schwannoma in patients with advanced hearing impairment.^236^

### Outcomes

#### Vertigo control

Case series and reviews indicate excellent outcomes, achieving 85%–100% complete or substantial control (AAO-HNS classes A/B) after well-executed labyrinthectomy. Failure or insufficient control is mainly related to residual vestibular epithelium, incorrect differential diagnosis, or actively progressive bilateral disease.^237^^,^^238^

#### Hearing

Classic labyrinthectomy results in ipsilateral anacusis. When combined with CI, consistent improvements in PTA, speech discrimination, and tinnitus complaints are observed, with hearing outcomes comparable to other classic CI indications.^236^

#### Quality of life

Substantial improvement in dizziness and overall disability is observed when vertigo control is achieved; older patients may compensate more slowly.^236^

### Complications and management

Facial nerve injury, cerebrospinal fluid leak, bleeding (sigmoid sinus), and infection

These complications are infrequent when the procedure is performed by experienced surgeons and become more common when the surgeon’s case volume is lower. As with other surgical complications, prevention depends on precise anatomic knowledge, careful hemostasis, and adequate sealing of the vestibule.^7^^,^^234^^,^^238^

Postoperative imbalance / Oscillopsia

This is an expected postoperative course after this type of procedure, given the underlying concept of the surgery. To minimize both discomfort and duration of symptoms, clinical management should be optimized with early vestibular rehabilitation and encouragement of mobilization.^233^

### Special populations and scenarios

#### Older adults

Vestibular compensation tends to be slower and more challenging in older adults. This should be considered during preoperative assessment and discussion of ablative treatment options. Intensive and appropriate vestibular rehabilitation reduces residual disability.^236^

#### Bilateral / progressive disease

Less ablative strategies should be preferred whenever possible because of the substantial functional impairment expected after radical labyrinthectomy. If labyrinthectomy is unavoidable ‒ for example, in refractory drop attacks unresponsive to less invasive management ‒ CI and realistic counseling regarding postoperative functional prognosis and quality of life should be planned.^234^

Recommendations for labyrinthectomy

LII Labyrinthectomy is recommended for patients with definite unilateral MD and disabling vertigo refractory to medical treatment and less invasive therapies (including intratympanic gentamicin and ESD), when hearing in the affected ear is poorly functional. (Strength of recommendation: moderate; Level of evidence: low).
LIII Before indicating any ablative procedure, the risk of progression to bilateral disease should be thoroughly assessed and discussed with the patient, considering that labyrinthectomy may result in severe functional impairment if contralateral disease develops. (Strength of recommendation: strong; Level of evidence: moderate).
LIV In patients with MD and no useful hearing, combined labyrinthectomy and CI (simultaneous or staged) should be considered, as it enables effective vertigo control associated with significant hearing gains and improved quality of life. (Strength of recommendation: moderate; Level of evidence: low).
LV The transmastoid route is the preferred approach for labyrinthectomy in MD, and complete extirpation of the vestibular epithelium (3 semicircular canals and vestibule) is essential to ensure sustained vertigo control and reduce the risk of treatment failure. (Strength of recommendation: weak; Level of evidence: low).
LVI After labyrinthectomy, early mobilization and structured vestibular rehabilitation, preferably initiated during the first postoperative week, are recommended to accelerate central vestibular compensation and reduce persistent imbalance, especially in older patients. (Strength of recommendation: weak; Level of evidence: low).

## New surgical approaches

The pathogenesis of MD remains unclear, and treatment is primarily aimed at controlling vertigo onset and delaying hearing loss progression. Consequently, MD management still lacks a targeted and systematic therapeutic approach, resulting in a considerable number of patients inevitably progressing from the early to late stages of the disease and eventually requiring surgical intervention. The primary goal of surgical treatment is vertigo control. Traditional surgical procedures for vertigo include endolymphatic sac surgery, VN, and labyrinthectomy. With the exception of endolymphatic sac surgery, the latter 2 procedures completely eliminate vestibular function. Endolymphatic sac surgery is the most popular surgical treatment for MD, although definitive evidence supporting its efficacy is lacking. Labyrinthectomy provides superior vertigo control but at the cost of complete hearing loss. VN achieves higher vertigo control rates but may lead to facial dysfunction and intracranial infection.^239^

Based on differing objectives, surgical methods for vertigo may be divided into 3 categories: surgical procedures targeting the potential causes and pathogenesis of vertigo, procedures aimed at obstructing the vestibular conduction pathway, and procedures focused on the reconstruction of vestibular and cochlear functions.^240^

Because MD is difficult to diagnose, exhibits substantial variability in presentation, and follows a fluctuating natural course, evaluation of any intervention is challenging and requires careful long-term follow-up. Assessment of treatment success should include the number and severity of acute vertigo attacks, hearing loss or gain, tinnitus severity, perception of aural fullness, symptom duration in relation to clinical improvement, and complications and adverse effects.

In recent years, concerns regarding patients’ quality of life after vertigo control have significantly altered the concept of surgical treatment of vertigo. Beyond controlling vertigo, there is increasing emphasis on preserving residual vestibular and cochlear function and even on reconstructing function whenever possible.^240^

In most patients with advanced MD, vestibular and auditory functions are severely impaired but not completely lost. Residual function remains highly important, particularly in patients who may progress from unilateral to bilateral disease. Therefore, preservation of vestibular function to the greatest extent possible is essential for maintaining postural balance. Furthermore, preservation of vestibular and cochlear end organs provides the basis and opportunity for future therapeutic advances.^241^

This section addresses semicircular canal plugging, which aims to ablate endolymphatic movement in the occluded canal; positive pressure therapy; and vestibular implant, which remains experimental in patients with bilateral vestibular dysfunction.

### Triple semicircular canal plugging

Triple Semicircular Canal Plugging (TSCP) mechanically obstructs the membranous semicircular canals to interrupt endolymph flow. In 1990, semicircular canal occlusion was first applied for the treatment of intractable BPPV.^242^ Since then, some investigators have explored whether semicircular canal occlusion could be used for the treatment of intractable peripheral vertigo, such as advanced MD (stage 4, mean hearing threshold >70 dB HL).^243^

The procedure is performed under general anesthesia via a retroauricular approach. All 3 bony semicircular canals are exposed through mastoidectomy. Bone drilling is performed until the “blue line” in the central portion of the canal is exposed. A 1–2 mm segment of the canal is skeletonized to create a fenestra, without opening the endosteum or membranous labyrinth. A plug of temporalis fascia is inserted through the fenestra to compress the endosteum and membranous labyrinth. The fenestration is sealed with bone wax to prevent perilymphatic fistula.^244^

With semicircular canal occlusion, endolymphatic fluid in this position is blocked and the movement of endolymph within a semicircular canal is substantially reduced, leading to minimal hair cell stimulation during angular motion and changes in endolymphatic pressure. TSCP requires precise localization of the semicircular canals, supported by high-resolution microsurgical instruments and intraoperative neurophysiologic monitoring (e.g., facial nerve integrity monitoring). These technical demands may limit feasibility in resource-constrained settings.^245^

Zhang et al.^243^ reported 97% vertigo control in 361 patients. However, 26.3% experienced postoperative hearing deterioration, emphasizing the need for careful risk-benefit analysis. The frequency of vertigo attacks 6-months before treatment was compared with the frequency of attacks occurring between 18- and 24-months after treatment. Caloric testing showed that surgery induced canal paresis on the affected side in all patients, which indicated that TSCP may control vertigo by eliminating semicircular canal function. VEMP results did not differ significantly before and after TSCP, suggesting preservation of otolithic organ function. Thus, vestibular compensation may occur more rapidly than after labyrinthectomy or VN. Postoperative imbalance resolved in most patients within 1–2 weeks of TSCP surgery, a shorter interval than typically observed after labyrinthectomy or VN.

Compared with intratympanic gentamicin, TSCP caused limited damage to semicircular canal and cochlear hair cells, acting primarily by blocking endolymphatic movement in the canals to control vertigo. Theoretically, TSCP was therefore less destructive than chemical labyrinthectomy. The rate of hearing loss was similar between TSCP and intratympanic gentamicin. The study demonstrated vertigo control comparable to VN or labyrinthectomy and superior to intratympanic gentamicin.^246^ Regarding the phenomenon in which all patients exhibit spontaneous nystagmus immediately after surgery and some patients complain of minor imbalance for several weeks, Zhang et al.^243^ attributed this to the “quiescent stage” of the end organs, in which resting discharge is temporarily affected but eventually recovers as the end organs remain morphologically intact. If the patient also has vestibular dysfunction in the contralateral ear, oscillopsia may occur after TSCP, which should be considered when selecting treatment.

A retrospective comparative study of 12 patients undergoing TSCP and 23 patients undergoing VN also demonstrated comparable efficacy in vertigo control.^247^ However, the TSCP group had significantly lower rates of postoperative paroxysmal dizziness and unsteadiness than the VN group (p = 0.020). DHI scores at 24-month follow-up showed superior quality-of-life outcomes in the TSCP group, with significantly lower scores than those in the VN group. It is plausible that the vestibular nerve on the healthy side cannot fully compensate for the balance disorders caused by VN on the affected side. The study also showed that DHI scores more than 24-months after TSCP were significantly lower than those within the first 24-months, reflecting improvements in patient emotional, physical, and functional quality of life. Because only stage 4 patients were included, hearing preservation or improvement could not be assessed.^247^ TSCP and VN both achieve high vertigo control rates. Compared with VN, TSCP offers several advantages, including less surgical trauma, faster recovery, and lower risk of facial nerve injury. While VN has proven to be effective in controlling vertigo, it is important to consider the potential risks and complications associated with the procedure, some of which can be life-threatening. In addition, the process of vestibular compensation following VN is more intricate and may prolong return to work.

Liu et al.^245^ followed 18 patients with treatment-refractory MD for a mean of 165-months. They observed that 77.78% (14/18) achieved vertigo control with the Meniett device (Medtronic Xomed, Jacksonville, FL, USA), but 4 patients required rescue therapy. ESD achieved control in 50% of failures, and subsequent TSCP successfully treated the remaining 2 cases. These findings support a stepwise surgical approach with long-term monitoring for MD unresponsive to medication.

The combination of ESD and TSCP showed 100% vertigo control in selected small-sample studies. ESD can potentially minimize inner ear damage, including aggravation of endolymphatic effusions, due to increased pressure in the long term resulting from plugging of semicircular canals. Combining ESD and TSCP seeks to mitigate the disadvantages of each procedure alone, aiming for higher success rates in controlling vertigo, while minimizing the risk of hearing loss and allowing a balance to be achieved between vertigo control and hearing preservation.^245^^,^^249^

Riemann et al.^247^ and Xiao et al.^248^ documented, in case reports, synergistic benefits of combining ESD/TSCP with CI in moderate-to-profound sensorineural hearing loss, including hearing restoration, vertigo control, and improved quality of life. For patients with unilateral MD and moderately severe-to-profound sensorineural hearing loss associated with frequent vertigo attacks, simultaneous CI, ESD, and TSCP proved to be an effective therapeutic strategy. The procedure is less traumatic than labyrinthectomy because hearing preservation remains possible. Preservation of otolithic organ function possibly leads to a better postural compensation and, in case of future involvement of the contralateral inner ear, it can provide an advantage for balance rehabilitation.

In a systematic review and meta-analysis evaluating the effects of TSCP on hearing in patients with MD, Zhu et al.^236^ reported hearing loss rates ranging from 7% to 36%, with a mean of 22.0%. Most patients had a hearing loss of 10–20 dB. Subgroup analyses revealed that disease duration and follow-up length had limited effects on postoperative hearing loss. Thus, patients undergoing TSCP have a better chance of preserving residual hearing compared with traditional surgery such as labyrinthectomy. The mechanisms underlying TSCP-related hearing loss remain unclear but may involve perilymphatic fistula, inner ear hemorrhage, rupture of the membranous labyrinth, serous labyrinthitis, or infection. The viability of the cochlea may be influenced by pressure changes in the membranous labyrinth during TSCP. Additionally, the study demonstrated vertigo control rates between 97% and 100%, consistent with previous reports, and highlighted the advantage of reducing chronic postoperative imbalance or exercise-induced vertigo compared with traditional surgical procedures, such as VN and labyrinthectomy.

Like ESD, TSCP may be considered a function-preserving surgical approach. Function-preserving techniques are increasingly favored because of their ability to mitigate vertigo while minimizing hearing damage. VN and labyrinthectomy, which carry risks of irreversible hearing loss and facial nerve dysfunction, are reserved for end-stage patients with irreversible hearing loss refractory to conservative and function-preserving surgical approaches.

Treatment outcomes among surgical modalities show marked heterogeneity. ESD achieves vertigo control rates of 65%–80%, although long-term recurrence remains high (18.2%–37.5%).^250^, ^251^, ^252^ In contrast, TSCP provides superior vertigo control (96.7%–100%) but is associated with a higher risk of postoperative hearing deterioration (22.9%–26.3%).^246^

This therapeutic dichotomy has driven the exploration of combined approaches, although the safety profiles of these innovative modalities still require validation through large-scale multicenter trials. Furthermore, surgeons proficient in both techniques are rare. In advanced MD, combined approaches (e.g., ESD + TSCP) may balance vertigo control (100%) with hearing preservation.^251^

The current literature on TSCP for MD treatment presents several limitations that deserve to be acknowledged.

First, available studies are based predominantly on retrospective analyses and case reports, with a scarcity of RCTs. This limitation inherently introduces selection bias, thereby restricting the reliability of causal inferences. Second, heterogeneity in therapeutic efficacy evaluation indicators (such as inconsistent definitions of “vertigo control” and lack of uniformity in audiologic assessment criteria) hinders comparability across studies and the effective integration of meta-analyses. Furthermore, TSCP lacks long-term follow-up data (>5-years).^246^^,^^247^ In addition, technical variability in surgical procedures (e.g., the choice of materials for plugging) and the lack of quantitative assessment of surgeon experience may introduce confounding effects in therapeutic efficacy evaluation. These limitations underscore the need for standardized reporting guidelines, multicenter RCTs with extended follow-up periods, and mechanistic studies exploring the relationship between surgical outcomes and objective biomarkers of inner ear dysfunction.

### Positive pressure therapy

Pressure changes in the middle ear can transmit to the inner ear through the round and oval windows. A transient increase in endolymphatic pressure has been suggested to promote the opening of a temporarily blocked endolymphatic duct or stimulate vascular regulatory mechanisms in the inner ear. Therefore, modulation of endolymphatic pressure has been proposed as a potentially beneficial therapeutic strategy for MD.^253^

Experimental studies in guinea pigs have demonstrated that intermittent positive pressure application reduces the development of endolymphatic hydrops.^254^ The technique is based on the generation of small pressure pulses by a pump attached to tubing placed in the ear canal. The placement of a ventilation tube in the tympanic membrane is required to ensure transmission of pressure pulses to the inner ear. In classic protocols, therapy is administered for approximately 5-minutes up to 3-times daily, although variations in frequency and duration have also been reported.^255^

The Meniett device is the principal device developed for this purpose. It consists of a portable unit connected to a probe inserted into the external auditory canal. The device generates small pressure pulses (0.4–1.2 kPa) at an approximate frequency of 6 Hz for 60-seconds, interspersed with 40-second pauses. The complete cycle lasts approximately 5-minutes and is usually repeated 3-times daily, 3-days per week, for at least 2-months. Protocol variations have also been described in the literature.

The theoretical advantage of this method is the ability to precisely control pulse parameters and directly transmit them via an opening in the tympanic membrane to the inner ear fluids without substantial energy dissipation. Because the endolymphatic system is relatively compressible, dynamic pressure change may induce fluid displacement and assist in compensating endolymphatic hydrops.^248^

Huang et al.^253^ reported positive effects on vertigo control and hearing improvement in 18 patients with intractable active MD treated with the Meniett device, with an initial follow-up of 2-years. In a subsequent extension study, these patients were followed for more than 13-years (mean 165-months), with a failure rate of 22.2%. The authors concluded that positive pressure therapy may provide sustained benefits in vertigo control, although long-term efficacy remains limited.

Conversely, Russo et al.^254^ published a multicenter, randomized, double-blind, placebo-controlled trial demonstrating symptom improvement in all treated patients, with no difference between the Meniett and placebo groups. However, follow-up was limited to 6-weeks, which is insufficient given the fluctuating and unpredictable natural course of MD.

Webster et al.^252^ conducted a systematic review of RCTs and concluded that positive pressure therapy may slightly increase the proportion of patients with improved vertigo frequency at 3–6 months. However, evidence on the actual change in vertigo frequency or global vertigo scores remains uncertain. No significant differences in quality of life or consistent effects on hearing or tinnitus were observed. The review also identified no serious adverse events associated with treatment. Overall evidence quality was considered low and uncertain.

Although positive pressure therapy with Meniett is a minimally invasive alternative, its clinical benefits remain controversial. Long-term studies suggest some positive impact on vertigo control, but short-term controlled trials have failed to demonstrate superiority over placebo. Methodological limitations, small sample sizes, and protocol heterogeneity reinforce the need for multicenter RCTs with extended follow-up to clearly define the role of this modality in MD management.

### Vestibular implant

Over the past 2 decades, the feasibility of a vestibular implant has been investigated. Animal and human studies have demonstrated that electrical stimulation may effectively activate the vestibular system. The principal groups involved in the research and development of this device are the Geneva-Maastricht group and the Washington University group.

The implant can provide motion information to the vestibular nerve through electrodes implanted in the labyrinth. It was designed to aid patients with bilateral vestibulopathy (bilateral vestibular hypofunction), a condition that can lead to a severe impairment of quality of life due to physical symptoms, such as imbalance and oscillopsia, as well as a negative impact on cognition and mood.^256^, ^257^, ^258^ Etiologies vary, but patients are most frequently diagnosed with genetic disorders, MD, ototoxicity, infectious diseases, and neurodegenerative diseases. However, in approximately one-third of patients, the etiology remains idiopathic.^259^

The vestibular implant consists of a modified CI, containing 1–3 “vestibular” electrodes implanted in the perilymphatic space adjacent to the membranous labyrinth of each semicircular canal.^260^ Therefore, rather than detecting sound information as the CI, it captures motion information using head-fixed sensors and transmits it to a special processor, where it is converted into an appropriate neural pattern. The neural patterns of processed motion information are then transmitted to the CNS in the form of electrical currents delivered via the vestibular electrodes to the implanted stimulator.^261^

In this concept, the vestibular implant seeks to transmit “artificial” neural patterns to the CNS similar to those coded by the normally functioning vestibular system.^261^ To date, 2 surgical strategies have been described for the vestibular implant: the intralabyrinthine and extralabyrinthine approaches. In the intralabyrinthine approach, each semicircular canal is opened, and electrodes are inserted until they contact the ampullary hair cells. In the extralabyrinthine approach, the labyrinth is not opened, and electrodes are placed directly onto the nerves.^262^

The intralabyrinthine approach is more commonly used, likely because the extralabyrinthine approach is surgically more challenging regarding the extent of the exact site of stimulation. However, the main disadvantage of the intralabyrinthine approach is the risk of hearing impairment when the labyrinth is opened, and the electrodes are inserted into the semicircular canals. For this reason, the Geneva-Maastricht group elected to perform surgery only in deaf patients in the implanted ear.^262^

Phillips et al.^260^ performed surgical implantation in 4 patients with MD and residual hearing; all subsequently lost hearing. However, these negative results do not necessarily imply that hearing will always be affected by electrode implantation in the semicircular canals. Studies in rhesus monkeys demonstrated that hearing preservation is possible with the intralabyrinthine approach.^263^, ^264^ Therefore, a major challenge for future research is optimization of the surgical technique and development of electrodes that can elicit effective stimulation for the activation of vestibular function while simultaneously preserving hearing.

After conducting a systematic review on vestibular implants, Azevedo et al.^259^ concluded that the analyzed studies demonstrated that electrical stimulation of the vestibular system through implants is capable of artificially restoring the Vestibulo-Ocular Reflex (VOR) in patients with bilateral vestibular loss, regardless of etiology or duration of deficit. The use of a virtual motion profile applied to the ampullary portions of the vestibular branch of the eighth cranial nerve effectively induced eye movements in all study patients. However, stimulation was associated with reduced visual acuity during use and with hearing and vestibular impairment in the implanted ear, especially in patients with MD. Thus, although restoration of the VOR represents a relevant functional gain, this benefit must be carefully weighed against the risks of residual hearing deterioration. Accumulated experience also indicates that human adaptation to vestibular electrical stimulation is feasible and occurs with acceptable levels of discomfort. Approximately 77% of implanted patients showed measurable improvement in vestibular function during follow-up. Another important aspect relates to dynamic response: the artificial VOR reached maximum performance at rotation frequencies of 1 and 2 Hz, which correspond to daily activities such as walking. Under these conditions, results may be regarded as a substantial functional recovery, even in patients with different etiologies and different vestibular deficit durations.^265^

Therefore, this technology appears promising for the rehabilitation of patients with bilateral vestibulopathy, although caution is recommended in cases of MD because of the risk of additional hearing loss. Beyond hearing deterioration, implanted patients with MD have also shown a decline in vestibular function and worsening response to electrode stimulation. This contrasts with promising results in rhesus monkeys, which maintained stable responses and minimal hearing loss. Investigators hypothesized that ears already damaged by MD may have reduced recovery capacity, or that more frequent stimulation in monkeys may have better preserved the vestibular nerve.^265^

Careful candidate selection, rigorous postoperative monitoring, and the development of protocols minimizing adverse effects, particularly auditory complications, are essential to broaden the clinical applicability of this intervention. TSCP represents an option for advanced MD refractory to medical treatment and may be considered an alternative to VN and labyrinthectomy, offering similar vertigo control with lower morbidity.

Recommendations for new surgical approaches

LVII TSCP may be considered a surgical option in patients with advanced MD refractory to medical treatment, showing high vertigo control rates (96%–100%) and lower postoperative morbidity compared with VN or labyrinthectomy. (Strength of recommendation: moderate; Level of evidence: moderate).
LVIII TSCP is associated with a relevant risk of postoperative hearing deterioration (20%–30%), although it provides greater preservation of otolithic organ function and faster vestibular compensation compared with classic ablative procedures. (Strength of recommendation: moderate; Level of evidence: moderate).
LIX Combned approaches, such as the combination of ESD and TSCP, may be considered in selected cases of advanced MD to balance vertigo control and hearing preservation, although validation in large-scale prospective studies is still lacking. (Strength of recommendation: weak; Level of evidence: low).

## Hearing rehabilitation

The treatment of MD is complex because hearing, vertigo control, tinnitus suppression, and quality of life are interrelated and therefore difficult to address in isolation. Most patients with MD will develop varying degrees of permanent hearing loss, and in 15%–38% of patients with unilateral MD, hearing loss will progress to severe sensorineural hearing loss.^266^ Bilateral MD rarely presents simultaneously. Most cases occur sequentially, with initial unilateral symptoms progressing to bilateral disease.^267^

### Hearing aids

Hearing fluctuation and tinnitus are frequent in patients with MD. Johns et al.^266^ described changes in low- and high-frequency thresholds and evident hearing fluctuation >10 dB in 27% of patients with MD during clinical follow-up. Most patients with MD seek treatment for their vestibular symptoms because these symptoms are the most distressing and life disruptive. Hearing loss has been often overlooked. In some cases, hearing aids are not considered an option for individuals with MD. Reasons for this include poor speech recognition scores in the ear affected by MD, unilateral hearing loss, and hearing fluctuation.^267^, ^268^, ^269^

The use of Personal Sound Amplification Products (PSAPs) is considered first-line rehabilitation in patients with MD who have mild-to-severe hearing loss and retain some functional hearing. Studies have demonstrated that amplification may enhance communication, reduce the social impact of hearing loss, and contribute to improved speech perception, although hearing fluctuation, recruitment, and the presence of low-frequency air-bone gaps may complicate fitting.^270^^,^^271^

Specific strategies include programming multiple settings for different hearing states, use of slow-acting compression, and features such as directional microphones and assistive listening devices to compensate for typical difficulties in noisy environments.^7^ In asymmetric or unilateral nonfunctional hearing loss, Contralateral Routing of Signal (CROS) devices or bone conduction systems may be useful, although they do not restore sound localization ability.^270^, ^271^, ^272^

### Cochlear implant

CI is indicated in patients with bilateral or unilateral MD who have severe-to-profound hearing loss and poor functional performance with PSAPs. A systematic review evaluated 17 studies demonstrating the safety and efficacy of CI in 182 patients with MD.^273^^,^^274^ Despite study heterogeneity, patients with MD experienced significant hearing improvement after CI and complication rates comparable to those observed in CI users without MD. Outcomes were similar to those in the general population of CI users. Only 3 patients (1.6%) had a decline in speech perception scores.

Improvement in vestibular symptoms after CI surgery has also been reported. Possible explanations for vestibular symptom resolution after CI placement include changes in inner ear fluid homeostasis or an inflammatory state with subsequent fibrosis caused by the presence of the electrode itself, which may alter endolymph flow in the semicircular canal. A study in guinea pigs demonstrated changes in the outflow of the endolymphatic system, with morphologic evidence on micro-CT and ECochG responses consistent with the presence of secondary endolymphatic hydrops during the first weeks after CI. This was attributed to the increased volume of scala media causing ballooning of the compliant Reissner’s membrane and downward displacement of the basilar membrane.^275^ The saccule, which is connected to the cochlea via the ductus reuniens, is nearest to the basal turn of the cochlea where damage from the electrode insertion may most likely occur. Histopathologic examination of human temporal bones with cochlear endolymphatic hydrops after CI showed the onset of cochlear hydrops in 88% of the saccules secondary to fibrosis of the ductus reuniens.^276^

Canale et al.^274^ showed improvement in vestibular function in patients with MD presenting with an active and disabling vestibular syndrome before surgery, which completely regressed after CI activation, even without labyrinthectomy. McRackan et al.^275^ evaluated 21 implanted patients with definite MD: 90% had bilateral disease and 28.6% (6-patients) had active vestibular symptoms. After CI, 5-patients reported a reduction in vestibular disease, 2-patients were able to discontinue medication, and only 1-patient reported no benefit from CI. Regarding hearing, the authors reported poorer results than those observed in the general implanted population, with better outcomes in patients with long-standing MD.

Intraoperative perilymph loss due to round window opening, leading to a reduction in scala tympani pressure and consequent downward displacement of the basilar membrane, could explain the resolution of hydropic vestibular symptoms after CI placement. However, this hypothesis does not explain the persistence of favorable long-term results once the cochlea is (presumably) sealed. In this regard, Frodlund et al.^276^ demonstrated that the cochleostomy approach and the use of straight electrodes can cause intracochlear trauma associated with the development of fibrosis of the ductus reuniens and, therefore, with changes in endolymphatic homeostasis. In addition, approximately 70% of the vestibular symptoms associated with MD resolve over the long term; therefore, the disappearance of vestibular attacks after CI in patients with disabling MD may also be considered part of the natural course of the disease.^277^

Improved gait and balance stability after CI may alter a previously uncompensated vestibular damage, inducing compensation, increased self-confidence, and improved spatial orientation.^278^ The placebo effect of the intervention should not be underestimated as a possible cause of symptom improvement. The influence of psychological factors in MD and the interactions between anxiety and recurrence of vertigo attacks are well recognized.^279^

However, patients with MD may represent a postoperative challenge because they typically experience fluctuating performance requiring device reprogramming.^273^^,^^280^ In patients with bilateral MD, the status of the contralateral vestibular system should be considered before CI, as surgery may result in additional vestibular damage.^274^

Another relevant aspect is tinnitus improvement, with suppression or significant relief after CI.^7^ Preservation of residual hearing is possible but less frequent than in other populations, and patients should be appropriately counseled.^280^^,^^281^ Shorter tinnitus duration before CI, tonal tinnitus, higher preoperative THI scores, and round-window electrode insertion correlate with a positive influence of CI on tinnitus suppression.^282^ Tinnitus represents only one aspect of CI candidacy in patients with MD and, in isolation, may not be a justifiable indication for CI.

Two distinct MD endotypes have been identified, characterized by specific endolymphatic sac pathologies: degeneration in the MD-dg group (approximately 75% of patients) and incomplete development in the MD-hp group (the remaining 25%).^29^^,^^283^ These 2 pathologies can be reliably distinguished and diagnosed in patients with clinical MD using temporal bone CT imaging combined with established radiologic surrogate markers, including ATVA. In a study evaluating 115 adult patients with MD and a history of unilateral or bilateral CI, the odds ratio for CI in a patient with MD-hp compared with a patient with MD-dg was 8.4 (95% CI 4.3–16.1).^284^ The MD-hp endotype, frequently associated with bilateral disease and early-age disease onset, is strongly linked to a higher likelihood of CI. Endotyping patients with MD based on endolymphatic sac pathology can effectively stratify their risk of severe hearing loss, guiding personalized audiologic follow-up and clinical decisions regarding potential CI.^284^

## Labyrinthectomy combined with cochlear implant

In patients with unilateral MD refractory to conservative medical and surgical management who already have nonfunctional hearing, combining labyrinthectomy with CI may provide simultaneous control of vertigo attacks and hearing rehabilitation.^285^^,^^286^ Case series and prospective studies indicate high vertigo control rates, as well as substantial gains in speech perception, sound localization, and quality of life.^287^ Ideally, electrode insertion should be performed during the same surgical stage as labyrinthectomy.^288^ Approximately one-third of patients undergoing labyrinthectomy develop cochlear ossification, which may preclude later electrode array insertion.^285^^,^^289^

Bilateral MD presents additional treatment challenges. Because labyrinthectomy eliminates both hearing and vestibular function, it is often contraindicated when a patient has only one hearing ear and/or bilateral MD due to the risk of causing a complete lack of vestibular afferents. Although bilateral disease is rare, reported rates range from 2% to 78%, and risk appears to increase with disease duration.^7^ CI has been associated with a postoperative reduction in tinnitus loudness and awareness in up to 93% of patients.^290^ Fewer studies, however, have reported that CI can worsen or even precipitate tinnitus, with rates ranging from 4% to 26%.^271^^,^^290^

Central auditory pathway reorganization occurring after unilateral deafness, which could theoretically exacerbate tinnitus, may be alleviated by providing auditory input to the deaf ear.^288^

## Vestibular neurectomy and cochlear implant

Retrosigmoid VN is less frequently performed as an alternative for refractory vertigo, although success rates are high (89%–96%).^288^ Hearing preservation (within 10 dB of the preoperative level) can be achieved in most patients.^287^

In a study evaluating quality of life in patients with MD receiving a CI, 3 participants had undergone a previous retrosigmoid VN on the ipsilateral implanted ear 15–29 years before CI. All of them initially preserved their hearing thresholds, but a slow progressive decline in hearing thresholds led to severe-to-profound hearing loss. These patients achieved hearing outcomes similar to those of the rest of the MD group, but they performed better in the subdomains “basic sound perception” (p = 0.038), “speech” (p = 0.005), “activity” (p = 0.038), and “social interactions” (p = 0.038).^29^°

### Active middle ear implants and bone conduction devices

Because of the risk of hearing deterioration and fluctuation, active middle ear implants are not recommended for patients with MD. In patients unable to use hearing aids because of chronic eczematous otitis, these devices should be reserved for selected cases.

Bone conduction devices may be indicated in cases of unilateral profound deafness in patients with MD, as the disease most commonly occurs unilaterally. It is important to assess the risk of developing disease in the contralateral ear, which could make future use of the prosthesis unfeasible.

Recommendations for hearing rehabilitation

LX PSAPs should be considered first-line hearing rehabilitation for patients with MD who have mild-to-moderate sensorineural hearing loss and preserved functional hearing, even in the presence of hearing fluctuation, provided that specific fitting strategies and continuous audiologic follow-up are implemented. (Strength of recommendation: strong; Level of evidence: moderate).
LXI In patients with MD using PSAPs, specific programming strategies are recommended, including multiple programs, slow-acting compression, directional microphones, and assistive listening devices, to mitigate the effects of hearing fluctuation, recruitment, and impaired speech perception in noise. (Strength of recommendation: strong; Level of evidence: low).
LXII CI should be offered to patients with unilateral or bilateral MD who have severe-to-profound sensorineural hearing loss and unsatisfactory functional performance with PSAPs, since CI is safe and effective, with hearing outcomes and complication rates comparable to those observed in other etiologies. (Strength of recommendation: strong; Level of evidence: moderate).
LXIII CI may be associated with improvement or partial resolution of vestibular symptoms and tinnitus in patients with MD, although these effects are not universal and may be related to the natural course of the disease, peripheral and central mechanisms, or placebo effects; these aspects should be clearly discussed with the patient preoperatively. (Strength of recommendation: moderate; Level of evidence: low).
LXIV In patients with unilateral MD, refractory vertigo, and nonfunctional hearing, combined labyrinthectomy and CI, preferably performed during the same surgical stage, may be considered for simultaneous control of vertigo attacks and hearing rehabilitation; the risk of cochlear ossification should be weighed if CI is not performed immediately. (Strength of recommendation: moderate; Level of evidence: low).
LXV Active middle ear implants are not routinely recommended for patients with MD because of fluctuating and progressive hearing loss. Bone conduction devices may be considered in selected cases of unilateral profound sensorineural hearing loss, with counseling regarding the risk of future contralateral involvement. (Strength of recommendation: weak; Level of evidence: low).

## Conclusion

MD should be understood as a complex, heterogeneous, and multifactorial clinical condition in which endolymphatic hydrops represents a central histopathologic finding, yet it is insufficient on its own to explain the broad clinical variability observed. Contemporary evidence supports the integrated involvement of genetic, immunologic, inflammatory, and inner ear ion homeostasis dysregulation mechanisms, reinforcing the concept of multiple phenotypes and endotypes with distinct diagnostic and prognostic implications.

The diagnosis remains fundamentally clinical, based on detailed medical history, validated international criteria, and serial audiometric documentation, while vestibular, electrophysiologic, laboratory, and imaging tests serve as complementary tools to be used judiciously and on an individualized basis. Accurate differentiation from alternative diagnoses, particularly in emergency settings and in the presence of common comorbidities, is essential to avoid inappropriate management and to optimize longitudinal patient follow-up.

The recommendations of this task force reflect expert consensus in light of the best available evidence, while acknowledging the current limitations of knowledge and the need for future research. Advances in molecular characterization, phenotypic stratification, and biomarker development may allow a more precise and personalized approach to MD, with direct implications for clinical practice, translational research, and patient quality of life.

## ORCID ID

Lucas Resende Lucinda Mangia: 0000-0003-3443-3640

Roberto Miquelino de Oliveira Beck: 0000-0003-3516-792X

Karen de Carvalho Lopes: 0000-0002-8888-0844

André Luiz Lopes Sampaio: 0000-0001-7611-1303

Patricia Mauro Mano: 0000-0002-5702-6039

Rogério Castro Borges de Carvalho: 0009-0005-0246-7362

Lucia Joffily: 0000-0002-8446-7083

Livia Nolêto de Rezende Oliveira: 0000-0003-0851-1123

Mauricio Noschang Lopes da Silva: 0000-0002-3747-5398

Vanessa Mazanek Santos: 0000-0003-4106-330X

Mariana Moreira de Castro Denaro: 0000-0003-4331-6438

Carlos Takahiro Chone: 0000-0002-4217-4629

## Funding

None.

## Data availability statement

The authors declare that all data are available in repository.

## Declaration of competing interest

The authors declare no conflicts of interest.
